# Functional Diversification of Hsp40: Distinct J-Protein Functional Requirements for Two Prions Allow for Chaperone-Dependent Prion Selection

**DOI:** 10.1371/journal.pgen.1004510

**Published:** 2014-07-24

**Authors:** Julia M. Harris, Phil P. Nguyen, Milan J. Patel, Zachary A. Sporn, Justin K. Hines

**Affiliations:** Department of Chemistry, Lafayette College, Easton, Pennsylvania, United States of America; The University of Arizona, United States of America

## Abstract

Yeast prions are heritable amyloid aggregates of functional yeast proteins; their propagation to subsequent cell generations is dependent upon fragmentation of prion protein aggregates by molecular chaperone proteins. Mounting evidence indicates the J-protein Sis1 may act as an amyloid specificity factor, recognizing prion and other amyloid aggregates and enabling Ssa and Hsp104 to act in prion fragmentation. Chaperone interactions with prions, however, can be affected by variations in amyloid-core structure resulting in distinct prion variants or ‘strains’. Our genetic analysis revealed that Sis1 domain requirements by distinct variants of [*PSI* ^+^] are strongly dependent upon overall variant stability. Notably, multiple strong [*PSI* ^+^] variants can be maintained by a minimal construct of Sis1 consisting of only the J-domain and glycine/phenylalanine-rich (G/F) region that was previously shown to be sufficient for cell viability and [*RNQ* ^+^] prion propagation. In contrast, weak [*PSI* ^+^] variants are lost under the same conditions but maintained by the expression of an Sis1 construct that lacks only the G/F region and cannot support [*RNQ* ^+^] propagation, revealing mutually exclusive requirements for Sis1 function between these two prions. Prion loss is not due to [*PSI* ^+^]-dependent toxicity or dependent upon a particular yeast genetic background. These observations necessitate that Sis1 must have at least two distinct functional roles that individual prions differentially require for propagation and which are localized to the glycine-rich domains of the Sis1. Based on these distinctions, Sis1 plasmid-shuffling in a [*PSI* ^+^]/[*RNQ* ^+^] strain permitted J-protein-dependent prion selection for either prion. We also found that, despite an initial report to the contrary, the human homolog of Sis1, Hdj1, is capable of [*PSI* ^+^] prion propagation in place of Sis1. This conservation of function is also prion-variant dependent, indicating that only one of the two Sis1-prion functions may have been maintained in eukaryotic chaperone evolution.

## Author Summary

Multiple neurodegenerative disorders such as Alzheimer's, Parkinson's and Creutzfeldt-Jakob disease are associated with the accumulation of fibrous protein aggregates collectively termed ‘amyloid.’ In the baker's yeast *Saccharomyces cerevisiae*, multiple proteins form intracellular amyloid aggregates known as yeast prions. Yeast prions minimally require a core set of chaperone proteins for stable propagation in yeast, including the J-protein Sis1, which appears to be required for the propagation of all yeast prions and functioning similarly in each case. Here we present evidence which challenges the notion of a universal function for Sis1 in prion propagation and asserts instead that Sis1's function in the maintenance of at least two prions, [*RNQ* ^+^] and [*PSI* ^+^], is distinct and mutually exclusive for some prion variants. We also find that the human homolog of Sis1, called Hdj1, has retained the ability to support some, but not all yeast prions, indicating a partial conservation of function. Because yeast chaperones have the ability to both bind and fragment amyloids *in vivo*, further investigations into these prion-specific properties of Sis1 and Hdj1 will likely lead to new insights into the biological management of protein misfolding.

## Introduction

Yeast prions are amyloid aggregates of functional yeast proteins that are both self-templating and heritable to daughter cells [1], [2], [3], [4]. The best studied yeast prion, called [*PSI^+^*], is the aggregated form of the yeast translation termination factor Sup35 [5], [6]. [*PSI^+^*] cells have a distinct phenotype characterized by enhanced nonsense suppression causing increased read-through of stop codons by translating ribosomes [7], [8], [9]. The phenotype arises from Sup35 sequestration in prion aggregates and the strength of the nonsense suppression correlates to the lack of soluble Sup35 [10]. Another yeast prion, first identified as the genetic element [*Pin*] for Psi inducibility, was later shown to be the aggregated form of the Rnq1 protein, hereafter called [*RNQ^+^*] for the high Asn (N) and Gln (Q) content of its prion forming domain [3], [11], [12]. Rnq1 is a cytosolic yeast protein of unknown function [4]. Neither the deletion nor overexpression of Rnq1, nor its aggregation in [*RNQ^+^*] cells results in any distinguishable phenotype beyond the tendency of [*RNQ^+^*] cells to spontaneously become [*PSI^+^*] at a greatly accelerated rate, hence the original denotation [*PIN^+^*] [3], [12], [13]. While both Rnq1 and Sup35 are cytosolic and have prion-forming domains, they do not significantly intermix in aggregates, and so [*RNQ^+^*] and [*PSI^+^*] form independent and stable structures *in vivo* [14].

Yeast prion propagation is dependent on the formation of heritable protein aggregates, often called ‘seeds’ or ‘propagons’, that can be passed on to daughter cells during cell division [15], [16]. Creation of yeast prion propagons results from the remodeling, and ultimately fragmentation, of amyloid aggregates by a specific set of cellular chaperone proteins minimally composed of Hsp70, Hsp104, and the J-protein Sis1, the focus of this study [17], [18], [19], [20], [21]. Sis1 functions as a co-chaperone protein with the Hsp70 Ssa in the fragmentation of at least four yeast prions ([*PSI* ^+^], [*RNQ* ^+^], [*URE3*], and [*SWI* ^+^]) and probably others [21], [22], [23], [24], [25]. Hsp70s, like the yeast protein Ssa, have two distinct domains for client-peptide binding and ATP hydrolysis [26]. The hydrolysis of ATP in one domain triggers a structural change in the other which enhances client peptide binding [27]. J-proteins, like Sis1, bind to the Hsp70 ATPase domain and catalyze ATP turnover, thus stimulating the association of Hsp70s with client peptides [28]. Because some J-proteins also bind client peptides themselves, they can also act as targeting factors, bringing Hsp70 to various cellular targets [29] [26]. Current models suggest that the J-protein Sis1 may act as a targeting factor which brings Ssa to prion aggregates, but the details of these interactions are unclear [21], [24], [26]. Hsp104, a disaggregase, is essential for the propagation of all known yeast prions [30], [31], [32]. The current model for chaperone-dependent prion fragmentation posits that following Sis1/Ssa intervention, which most likely results in a partial unfolding of a prion protein, the chaperone Hsp104 binds a free end or loop of the prion protein and feeds the full protein through its central cavity [33] [21], [23]. Multiple rounds of Hsp104-dependent monomer unfolding likely destabilize the aggregates, resulting in their eventual fragmentation [19].

**Figure 1:**
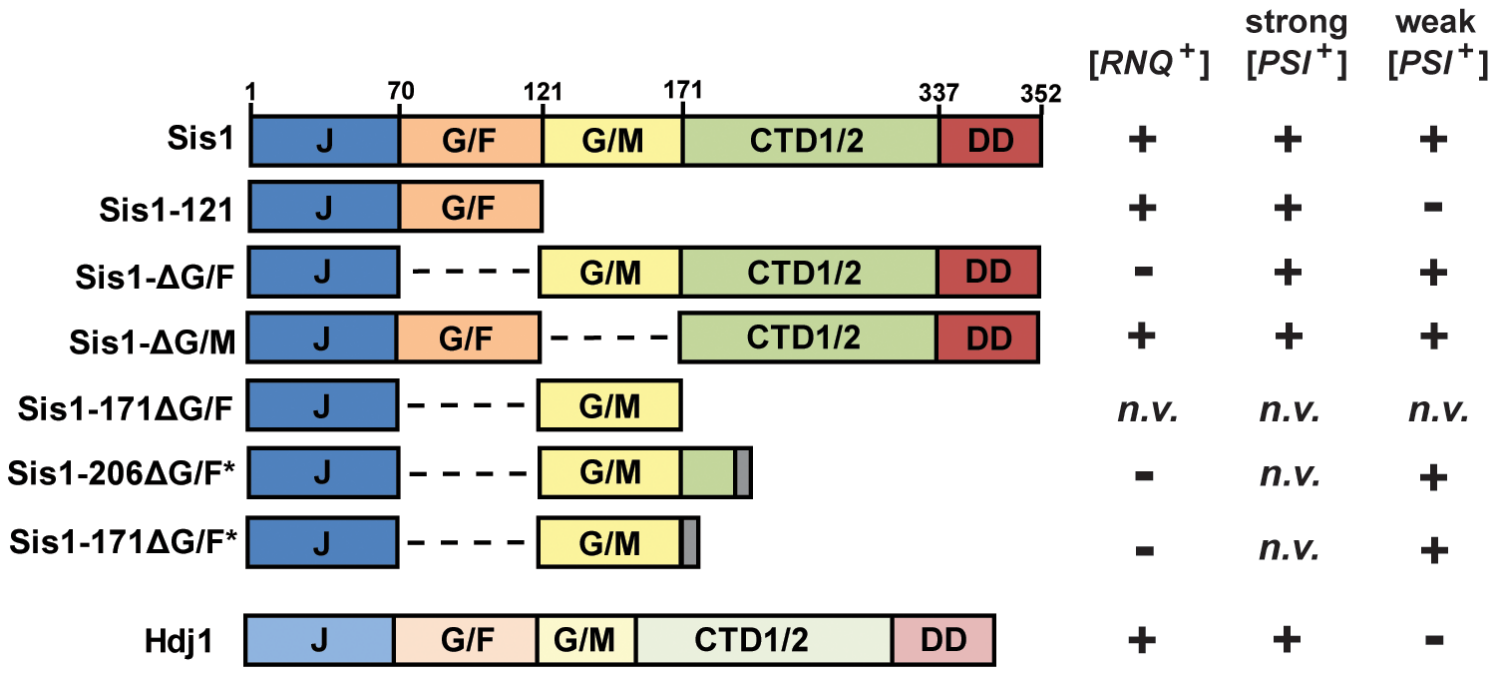
Constructs of Sis1 used in plasmid-shuffling experiments. (*left*) Gene structure diagrams of Sis1 and Hdj1 (*DNAJB1*) expression constructs. Gene regions are denoted using the following notation: *J*, J-domain; *G/F*, glycine/phenylalanine-rich region; *G/M*, glycine/methionine-rich region; *CTD1/2*, C-terminal peptide-binding domains I and II; *DD*, dimerization domains. Dashed lines indicate where a region had been deleted; gray bars represent a C-terminal tag. (*right*) A summary of the genetic interactions either previously known or uncovered in this investigation. Prion maintenance and loss are represented by plus and minus signs, respectively; *n.v.* indicates that the construct does not support cell viability.

Like other yeast J-proteins, Sis1 also has a domain-type architecture (**Figure 1**) including an N-terminal J-domain, which is critical for stimulation of Hsp70's ATPase activity, and C-terminal domains which are known to bind model peptides and are homologous to other known J-protein peptide-binding sites [34] [26], [35]. Separating the J-domain and C-terminal domains are two glycine-rich regions known as G/F (Gly and Phe rich) and G/M (Gly and Met rich). Sis1 has recently been implicated in spatial protein quality control pathways involving target-protein ubiquitylation and protein sorting [36] [37], [38], [39]. While its specific cellular functions are still unclear, Sis1 is also an essential yeast protein; yeast cells are inviable unless the J-domain of Sis1 is expressed in *cis* with at least one of these two glycine-rich regions, underscoring their biological importance [40]. However, despite being essential, Sis1's expression can be greatly reduced, or the protein may be truncated, resulting in the support of cell viability but not prion propagation [18] [23], [24], [25], [41], [42], [43]. For example, the C-terminal peptide-binding domain is generally dispensable for both viability and prion propagation [40] [41], [43]. In contrast, when the G/F region is absent (Sis1-ΔG/F, **Figure 1**), [*PSI* ^+^] is maintained but [*RNQ^+^*] is no longer supported [41] [24]. A construct of Sis1 which supports viability but not [*PSI* ^+^] propagation has never before been identified.

Due to a distinctive color phenotype, high mitotic stability, and a plethora of knowledge gleaned from years of investigations, the prion [*PSI* ^+^] has become the best understood and arguably, the model yeast prion against which others are often compared [44], but the behavior of a yeast prion *in vivo* can depend on multiple factors. Distinct prion ‘variants’ can result from the prion-forming protein's assumption of multiple amyloid conformations, which have distinct heritable characteristics [5] [45], [46], [47], [48]. Prion variants are typically classified as “strong” or “weak” based on phenotypic strength and mitotic stability, and often such variants have distinct chaperone requirements [42], [49] [47], [50]. Indeed, multiple variants of [*PSI* ^+^] are well-studied and described in the literature [5] [45], [46], [49]. Likewise, yeast genetic background can also affect the results of prion-chaperone experiments [18] [42]. Here we explore the requirement of Sis1 domains in the maintenance of [*PSI^+^*], with special care to explore the potential effects of yeast genetic background and yeast prion structural variability.

In a previous investigation, one of us (JKH) found that both the weak and strong [*PSI* ^+^] variants [*PSI* ^+^]^Sc4^ and [*PSI* ^+^]^Sc37^ could be maintained by Sis1-ΔG/F, demonstrating that [*PSI* ^+^] does not share the absolute requirement for Sis1's G/F domain with [*RNQ* ^+^] [24]. In a separate investigation, Kirkland *et al.* found that the regions of Sis1 known to be necessary to support cell viability (minimally Sis1-121) are also sufficient to support the propagation of a strong [*PSI* ^+^] variant [43]. To further investigate the ability of Sis1 to support [*PSI* ^+^] propagation, and to determine the influence of prion-variant structure and yeast genetic background on this model system, we first investigated the Sis1 requirements of well-characterized strong and weak [*PSI* ^+^] variants using yeast plasmid shuffling. Utilizing two distinct yeast genetic backgrounds, this approach, in combination with biochemical assays, revealed that Sis1 requirements are consistent between yeast genetic backgrounds and consistent among prion variants of similar mitotic stability (‘strength’) but differ greatly between weak and strong variants. Sis1-ΔG/F, which cannot maintain the prion [*RNQ* ^+^], maintains all variants of [*PSI^+^*] whereas Sis1-121, which supports [*RNQ* ^+^] propagation, cannot support weak [*PSI* ^+^] variants. Likewise the human homolog of Sis1, Hdj1, which supports [*RNQ* ^+^], is here shown to be capable of strong but not weak [*PSI* ^+^] variant propagation. This mutually exclusive set of chaperone requirements for by [*RNQ* ^+^] and weak [*PSI* ^+^] cannot be rectified by positing that the two prions simply have different levels of stringency for a singular Sis1 function. Rather, these data indicate that Sis1 must have at least two distinct functions in yeast prion maintenance which are prion specific and allow for J-protein-dependent prion selection.

## Results

### [*PSI* ^+^] requirements for Sis1 are strongly dependent upon prion amyloid structure, *i.e.*, prion-variant ‘strength’

**Figure 2:**
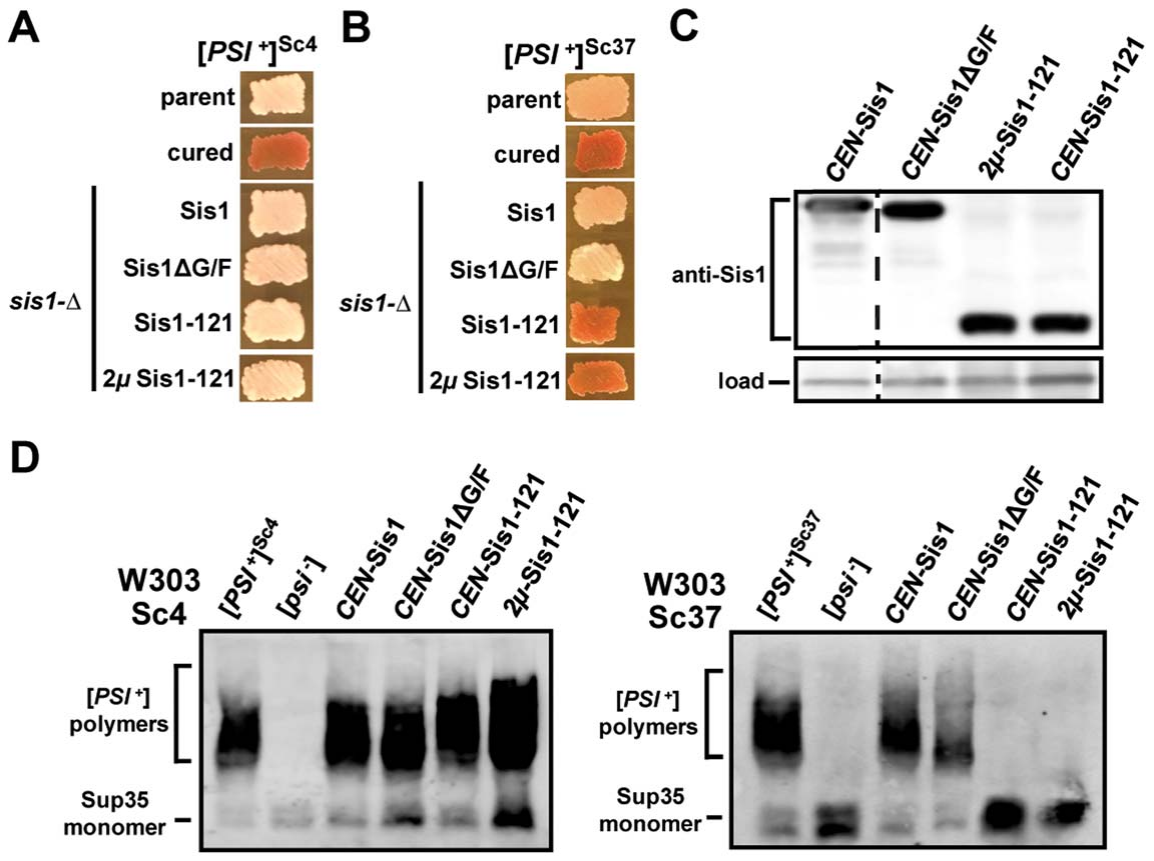
[*PSI* ^+^] requirements for Sis1 are prion-variant dependent. (A and B) Sis1-plasmid shuffling experiments to examine the potential effects of Sis1 domain deletions on [*PSI* ^+^] propagation. [*PSI* ^+^] bearing cells were transformed by plasmids expressing Sis1 truncations or deletions and subjected to plasmid shuffling. Color phenotype assays are shown for representative transformants (*n*≥6 for each plasmid) following loss of the [*SIS1*-Sis1, *URA3*] plasmid and bearing either the strong [*PSI^+^*] variant [*PSI^+^*]^Sc4^ (A), or the weak [*PSI^+^*] variant [*PSI^+^*]^Sc37^ (B). Parental [*PSI* ^+^] cells for each variant (parent) and cells cured by growth in the presence of GdnHCl (cured) are included as positive and negative controls for colony color. Cells expressing full-length Sis1 (Sis1) from a plasmid were used as a positive control for the stability of the prion throughout the plasmid-shuffling procedure. For clarity, images taken from different parts of the same plate have been arranged in columns. (C) Sis1 protein expression levels in cells containing the weak [*PSI^+^*] variant [*PSI^+^*]^Sc37^ in the W303 genetic background. Cell extracts from isolates in panel B were subjected to immunoblot analysis using antibody specific for Sis1. A band cross-reacting with the Sis1 antibody is shown as a loading control. Dotted lines separate lanes taken from different parts of the same gel. (D) Maintenance or loss of [*PSI* ^+^] in cells shown in (A) ([*PSI^+^*]^Sc4^, *left*) and (B) ([*PSI^+^*]^Sc37^, *right*) was also confirmed by semi-denaturing detergent agarose gel electrophoresis (SDDAGE). Detergent resistant Sup35 aggregates indicative of the presence of [*PSI* ^+^] were resolved by SDDAGE and visualized by immunoblot analysis using an antibody specific for Sup35. Control [*PSI* ^+^] and [*psi* ^−^] cells for each variant were included for comparison.

To begin to examine the impact of amyloid variation on Sis1-domain requirements, we first determined the ability of two commonly used Sis1 protein constructs lacking key domains (Sis1-121 and Sis1-ΔG/F) to propagate the well-studied strong [*PSI* ^+^] variant [*PSI* ^+^]^Sc4^ by yeast plasmid shuffling [49] [46]. The plasmid shuffling procedure was the same as previously described (see Materials and Methods) [42] [25]. All strains remained [*PSI* ^+^] as indicated by white/pink colony color as compared to the [*PSI* ^+^] parent-strain (pink) and cured [*psi* ^−^] strain controls (red) (**Figure 2A**). These results confirm the combined observations of Higurashi *et al.* and Kirkland *et al.* that strong [*PSI* ^+^] can be maintained by either Sis1-121 or Sis1-ΔG/F [24] [43]. To next determine if Sis1 domain requirements are altered when the [*PSI* ^+^] prion is an alternate amyloid structure, *i.e.*, an alternate prion variant, we next examined the Sis1 requirements of [*PSI* ^+^]^Sc37^, a well-characterized weak variant (**Figure 2B**) [49] [46]. [*PSI* ^+^]^Sc37^ was maintained by the Sis1-ΔG/F construct, but, in contrast to the stronger variant, the prion was lost when Sis1-121 is the only version of Sis1 expressed in the cell. These results are surprising for two reasons: first, they are in direct opposition to the requirements for Sis1 by [*RNQ* ^+^], that is, [*RNQ* ^+^] is maintained by Sis1-121 but not Sis1-ΔG/F and second, because [*PSI* ^+^] has been previously thought to less-stringently require Sis1 activity for prion propagation when compared to [*RNQ* ^+^] and other prions [18] [25], [41], [42]. Previous rationalizations regarding the distinctions between [*PSI* ^+^] and [*RNQ* ^+^] in terms of Sis1 requirement based on Sis1-repression experiments have posited that [*PSI* ^+^] requires less Sis1 activity than [*RNQ* ^+^] [24]. The ability of Sis1-121, but not Sis1-ΔG/F, to maintain [*RNQ* ^+^] implies that the former construct is somehow more active than the latter. If valid, these new observations regarding [*PSI* ^+^]^Sc37^ would negate this model, as the two prions, [*RNQ* ^+^] and [*PSI* ^+^]^Sc37^, appear to have mutually exclusive requirements of Sis1.

To confirm that prion loss is due to the inability of Sis1-121 to support the prion, rather than due to a low level of protein expression, we next subjected these strains to immunoblotting using a Sis1 antibody which recognizes all three constructs (**Figure 2C**) [42]. Sis1-121 protein is expressed to level which is equal to or greater than the wild-type protein, indicating that the loss of [*PSI* ^+^]^Sc37^ in these cells was not due to abnormally low Sis1-121 protein expression. Sis1 expression is subject to tight auto-regulation, even when expressed from an exogenous promoter, making it difficult to significantly over-express in yeast [42] [51]. In an effort to produce a higher level of Sis1 expression, we included in our experiment a *2μ* plasmid expressing Sis1-121 (*p324SIS1-sis1-121*) that produces slightly higher levels of expression than our *CEN* plasmid (*p314SIS1-sis1-121*) [42]. Results for this plasmid were the same, Sis1-121 supported the strong [*PSI* ^+^] variant [*PSI* ^+^]^Sc4^ but not the weak variant [*PSI* ^+^]^Sc37^ (**Figure 2A and 2B**) despite being expressed at a modestly higher level when compared to the loading control (**Figure 2C**).

To confirm that colony color is accurately reporting prion maintenance or loss in our strains, we next verified our results using an additional biochemical assay, semi-denaturing detergent agarose gel electrophoresis (SDDAGE), in which large detergent-resistant aggregates may be resolved using an agarose gel and then visualized by immunoblotting [52]. In all cases SDDAGE analysis confirmed our colony color observations: Sis1-ΔG/F maintained both variants while only the [*PSI* ^+^]^Sc37^ variant was lost when Sis1-121 is the sole form of Sis1 expressed (**Figure 2D**). Additionally, no drastic aggregate-size shifts were apparent in these samples.

**Figure 3:**
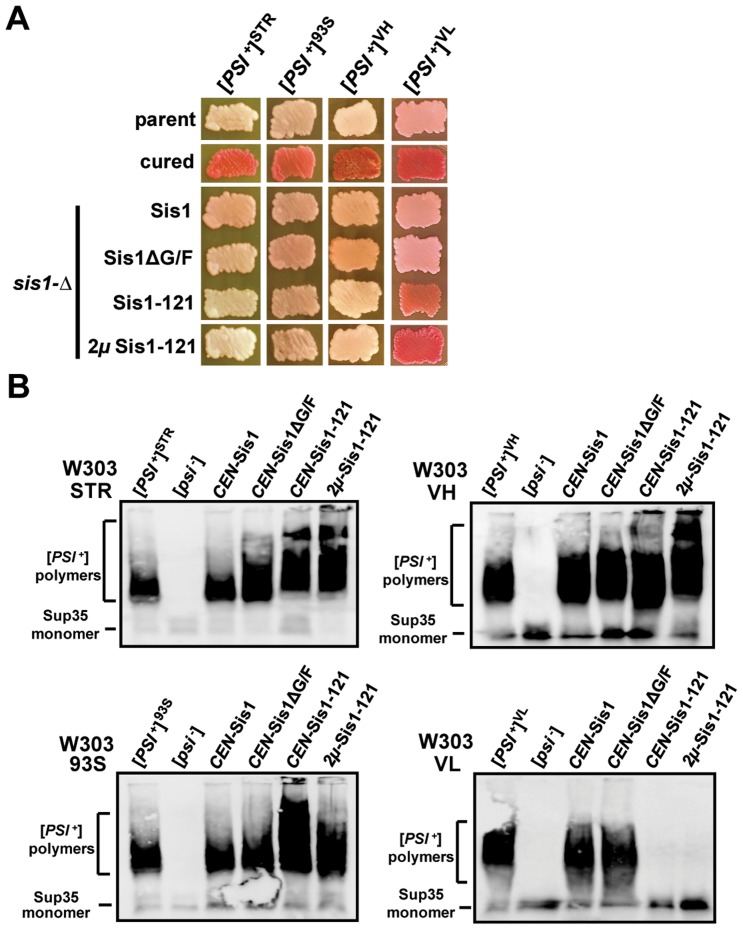
[*PSI* ^+^] requirements for Sis1 are strongly dependent upon overall variant strength. (A) [*PSI* ^+^] cells of the W303 genetic background bearing either strong [*PSI* ^+^] variants ([*PSI* ^+^]^STR^, [*PSI* ^+^]^VH^, and [*PSI* ^+^]^93S^) or the weak [*PSI* ^+^] variant [*PSI* ^+^]^VL^ were transformed by plasmids expressing Sis1 truncations or deletions and subjected to plasmid shuffling. Color phenotype assays are shown for representative transformants (*n*≥6 for each plasmid) following loss of the [*SIS1*-Sis1, *URA3*] plasmid. Parental [*PSI* ^+^] cells for each variant (parent) and cells cured by growth in the presence of GdnHCl (cured) are included as positive and negative controls for colony color. Cells expressing full-length Sis1 (Sis1) from a plasmid were used as a positive control for the stability of the prion throughout the plasmid-shuffling procedure. For clarity, images taken from different parts of the same plate have been arranged in columns. (B) Maintenance or loss of [*PSI* ^+^] in cells shown in (A) was also confirmed by semi-denaturing detergent agarose gel electrophoresis (SDDAGE). Detergent resistant Sup35 aggregates indicative of the presence of [*PSI* ^+^] were resolved by SDDAGE and visualized by immunoblot analysis using an antibody specific for Sup35. Control [*PSI* ^+^] and [*psi* ^−^] cells for each variant were included for comparison.

We next considered whether this unusual pattern of Sis1-domain requirement was specific to the individual weak [*PSI* ^+^] variant examined. In a prior investigation in which wild-type Sis1 expression was chemically repressed, large variations in curing rates were found between weak [*PSI* ^+^] variants, including [*PSI* ^+^]^Sc37^, in the W303 genetic background, indicating that there are differences between weak variants with regard to their interactions with Sis1 [42]. To determine whether the Sis1 requirements of [*PSI* ^+^]^Sc37^ are specific to this variant only, or are shared by other weak [*PSI* ^+^] variants, we expanded our investigation to include additional strong and weak variants of [*PSI* ^+^] in the W303 genetic background using the same plasmid shuffling approach described above. Specifically, the Sis1 domain requirements for three additional strong variants ([*PSI* ^+^]^STR^, [*PSI* ^+^]^VH^, and [*PSI* ^+^]^93S^) and one additional weak variant ([*PSI* ^+^]^VL^) were examined [42]. Colony color assays (**Figure 3A**) as well as SDDAGE analyses (**Figure 3B**) indicated that all three strong variants were maintained by all Sis1 constructs examined, whereas [*PSI* ^+^]^VL^ was lost specifically when Sis1-121 was the sole Sis1 construct expressed. These results support those already obtained here for strong and weak variants [*PSI* ^+^]^Sc4^ and [*PSI* ^+^]^Sc37^, respectively, and indicate that the minimal requirements for Sis1 function of strong variant [*PSI* ^+^]^Sc4^, and the unusual requirement for Sis1 of the weak variant [*PSI* ^+^]^Sc37^, are not peculiarities of these two particular variants, nor are they dictated by subtle difference in variants. Rather, they appear to be primarily determined by prion-variant strength, a property that arises from gross differences in amyloid core structure.

Finally, we also considered whether the maintenance of weak [*PSI* ^+^] variants by Sis1-ΔG/F here could be due to the unexpected expression of wild-type Sis1 in these strains, as homologous recombination rates are high *S. cerevisiae* and cross-over may occasionally occur during plasmid-shuffling during the period when both full-length and variant copies of Sis1 are present within a given cell. Immunoblotting with a Sis1 antibody confirmed that only Sis1-ΔG/F, not full-length Sis1, is expressed in these samples (**Figure S1**).

### [*PSI* ^+^] variant-specific Sis1 requirements are consistent across two distinct yeast genetic backgrounds

As noted in the introduction above, a common limitation of investigations in *S. cerevisiae* is that, for practical purposes, observations are rarely confirmed in more than one yeast genetic background, leaving open the possibility that polymorphisms of a particular yeast strain may affect the experimental outcomes and interpretations [42] [18], [44]. Indeed, incongruencies in observations of prion-chaperone interactions have been attributable to yeast strain variations in the past [18] [42]. To directly address this issue, we took advantage of a series of yeast strains used in a previous investigation which uncovered peculiar distinctions in the behavior of weak prion variants upon Sis1 repression between two different genetic backgrounds, W303 and 74D-694. These distinctions indicate that some still unidentified factors which differ between these two genetic backgrounds affect prion behavior *in vivo* [42]. To ensure that any results are not due to a peculiarity of the W303 yeast genetic background we reconstructed all of our [*PSI* ^+^] Sis1-plasmid shuffling strains in the 74D-694 genetic background and reexamined the Sis1 domain requirements for all of the variants described above. The results were summarily consistent with those obtained in the W303 background (**Figure S2**), reaffirming our initial observations and confirming that the unusual Sis1 requirements exhibited by [*PSI* ^+^]^Sc37^ and other weak variants are not due to any specific factor of the W303 genetic background, but are rather determined primarily by the strength of the [*PSI* ^+^] variant.

### Observed prion curing is consistent with prion loss rather than cell selection by [*PSI* ^+^]-induced cytotoxicity

[*PSI* ^+^] cells exhibit a slow growth phenotype when Sis1 expression is chemically repressed (*unpublished observations*) or when Sis1 is ectopically expressed in the form of C-terminal truncation mutants, a phenomenon which has been interpreted as a [*PSI* ^+^]-dependent toxicity against which Sis1 protects cells [43]. As such, the appearance of [*psi* ^−^] cells in the experiments described herein could be explained either as the inability of a particular Sis1 construct to support prion propagation, or as the result of an induced selection for [*psi* ^−^] cells as [*PSI* ^+^] cells become sick. In a previous investigation, others demonstrated that cytotoxicity is not due to reductions in Sup35, Sup45 or decreased Sis1 levels in the soluble fraction and suggested that toxicity is correlated to prion propagon number because toxicity diminishes as propagon number is decreased during Hsp104 inhibition by GdnHCl [43]. Indeed, the phenotype appears to be specific to [*PSI* ^+^], the yeast prion with the highest known number of heritable prion propagons per cell [44] [24], [25]. If true, then the patterns of prion curing that we have observed in this investigation are inconsistent with a [*psi* ^−^] cell selection model, since prion curing has occurred only in strains bearing weak [*PSI* ^+^] variants that are known to have fewer prion propagons than strong variants [5] [24], [46].

To further explore this phenomenon and to clarify our interpretations, we investigated whether strong or weak [*PSI* ^+^] variants which are known to differ in prion propagon number exhibit differential cytotoxicity upon Sis1 repression. To do this, we first set out to estimate the relative propagon numbers of the variants used in this study by conducting a propagon counting assay [53]. Each variant was tested in quadruplicate in the W303 genetic background. All four strong [*PSI* ^+^] variants produced curing data which was overlapping and fit a model with approximately 300 propagons/cell (**Figure S3**); as such, we were unable to distinguish between these variants on the basis of these data, consistent with previous observations that distinct strong [*PSI* ^+^] variants are cured with virtually identical kinetics upon Sis1 repression indicating that strong [*PSI* ^+^] variants may have converged on a similar, and perhaps optimum, amyloid structure for stable propagation in yeast [42]. In contrast, [*PSI* ^+^]^Sc37^ and [*PSI* ^+^]^VL^ curing data produced estimates of 90 and 75 propagons/cell, respectively. These numbers are in general agreement with previous estimates made for [*PSI* ^+^]^STR^, and [*PSI* ^+^]^Sc37^ using the same methods and genetic background [24], although it is worth noting that this method, while useful for drawing comparisons among prions, likely systematically underestimates the actual number of heritable propagons [54]. Next, we utilized a set of tetracycline-repressible strains which have been used previously to study Sis1•[*PSI* ^+^] genetic interactions [24] [42]. These strains have *SIS1* under the control of the tetracycline repressible (*TETr*) promoter (*sis1-Δ::LEU2* [*TETrSIS1*]). Following the addition of the tetracycline analog doxycycline, Sis1 expression is reduced, leading to eventual prion curing [42] [24]. Four W303 strains, each bearing a different [*PSI* ^+^] variant ([*PSI* ^+^]^Sc4^, [*PSI* ^+^]^Sc37^, [*PSI* ^+^]^VH^, or [*PSI* ^+^]^VL^) were cultured in log phase in rich media with similar growth rates, measured as averages of periods of approximately 8–10 generations, of 1.4–1.7 hrs/gen. Following the addition of doxycycline, all four strains experienced a slowing of growth rate, consistent with both reduced Sis1 levels and [*PSI* ^+^]-dependent toxicity. However, as predicted, the decline in growth rate was more dramatic for both of the strong [*PSI* ^+^] bearing strains, declining to 4.5 hrs./gen. for [*PSI* ^+^]^Sc4^ and 3.1 hrs./gen. for [*PSI* ^+^]^VH^ before beginning to recover slightly, than for the weak variant-bearing strains (slowest observed rates were 2.1 hrs./gen. and 2.2 hrs./gen. for [*PSI* ^+^]^Sc37^ and [*PSI* ^+^]^VL^, respectively). Cured versions were also examined as a control and exhibited no differences in growth rate among the four strains, indicating that these discrepancies are indeed caused by differences between strong and weak prion variants. These observations confirm the hypothesis forwarded by Kirkland *et al.* that [*PSI* ^+^] induced toxicity correlates with propagon number [43]. They also support the conclusion that the curing of weak [*PSI* ^+^] variants but not strong variants in this study is not due to cell selection by [*PSI* ^+^]-dependent cytotoxicity, but rather, by an inability of these chaperone constructs to support the propagation of the prion.

### Hdj1, the human homolog of Sis1, maintains strong but not weak [*PSI* ^+^] variants

**Figure 4:**
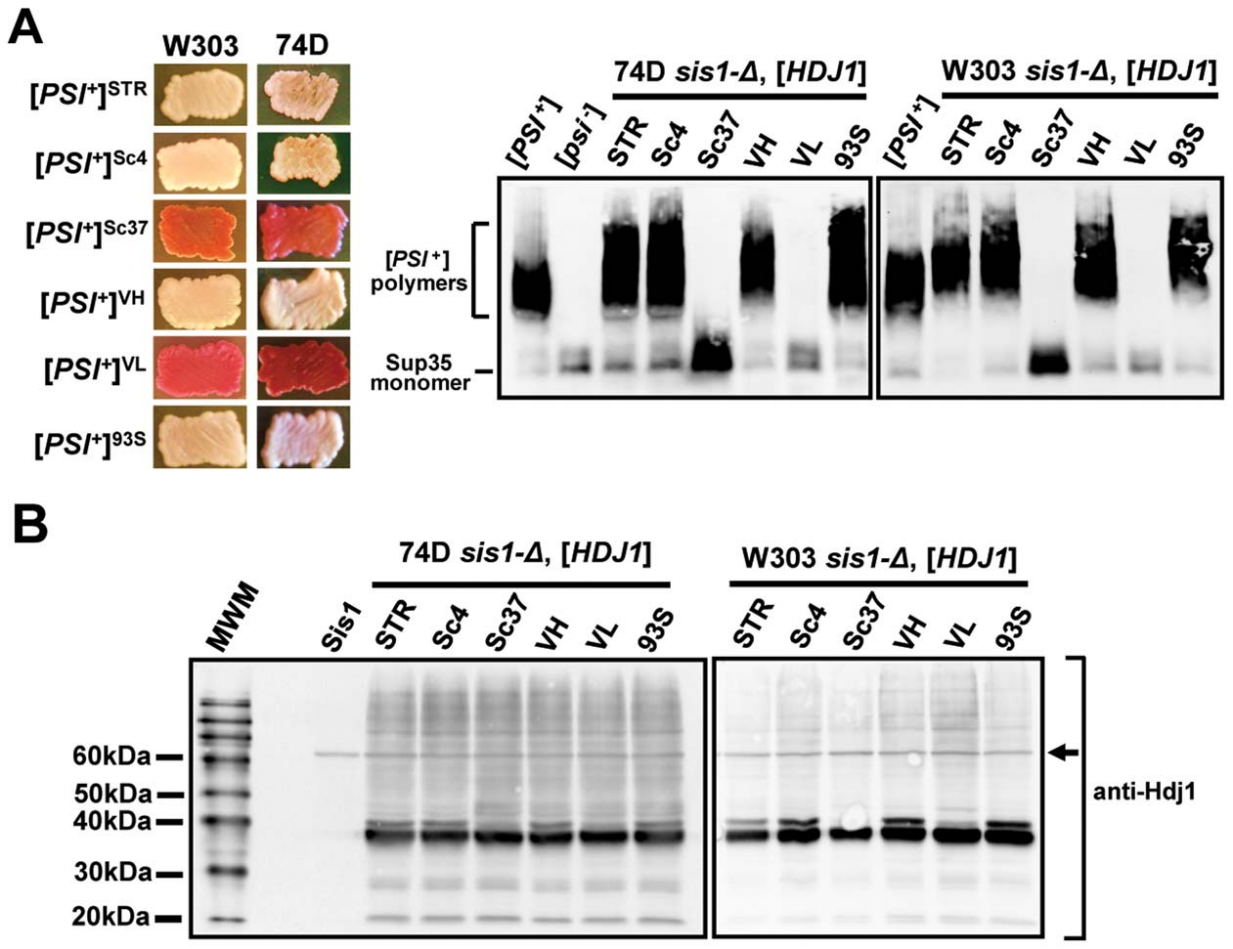
Hdj1, the human homolog of Sis1, maintains strong but not weak [*PSI^+^*] variants in two yeast genetic backgrounds. (A) *sis1-Δ* cells from two backgrounds (W303 and 74D-694) bearing each of the six variants of [*PSI* ^+^] examined in this study were transformed by a plasmid expressing Hdj1 from a constitutive promoter (*p424*-*GPD*-Hdj1) and subjected to plasmid shuffling to remove the covering Sis1-expression plasmid (*p316-SIS1-Sis1*). Color phenotype assays (*left*) are shown for representative transformants (*n*≥6 for each strain) following loss of the [*SIS1*-Sis1, *URA3*] plasmid. Maintenance or loss of each [*PSI* ^+^] variant in these cells was also confirmed by semi-denaturing detergent agarose gel electrophoresis (SDDAGE) (*right*). Detergent resistant Sup35 aggregates indicative of the presence of [*PSI* ^+^] were resolved by SDDAGE and visualized by immunoblot analysis using an antibody specific for Sup35. Control [*PSI* ^+^] and [*psi* ^−^] cells were included for comparison. (B) Lysates of all cells from (A) were resolved by SDS-PAGE and visualized by immunoblotting with Hdj1-specific antibodies that do not cross-react with Sis1. A lysate of a strain expressing only full-length Sis1 is included as a control. A band cross-reacting with the Hdj1 antibody in all lanes is shown as a loading control (*black arrow*).

The human protein Hdj1 (DNAJB1) shares >36% of residue identities with Sis1 (ALIGN), indicating that it is a Sis1 ortholog and that the two proteins likely share similar overall folds [55]. Indeed, Hdj1 can substitute for Sis1 in maintaining yeast cell viability with no obvious phenotypic differences, and can support the maintenance of the [*RNQ* ^+^] prion in cells otherwise lacking Sis1 expression [41]. Another investigation found that Hdj1 was incapable of supporting [*PSI* ^+^], though this investigation examined only one strong variant and experiments were conducted in a different yeast genetic background than utilized previously for [*RNQ* ^+^] [43]. In order to more broadly examine the ability of Hdj1 to maintain prions in yeast, we tested Hdj1 expression under the control of the *GPD* promoter from a *2μ* plasmid in all of our aforementioned Sis1-plasmid shuffling strains. Surprisingly, we found that Hdj1 was able to support all strong variants of [*PSI* ^+^] examined, in both the W303 or 74D-694 genetic backgrounds, as confirmed by both colony color and SDDAGE (**Figure 4A**). In contrast, Hdj1 was unable to maintain any weak variant of [*PSI* ^+^], similar to the pattern of [*PSI* ^+^] prion maintenance exhibited by Sis1-121. Notably, some size shifts in aggregate bands were apparent for strong [*PSI* ^+^] variants (**Figure 4A**). Prion aggregates maintained by Sis1 migrated further into the gel than those maintained by Hdj1, indicating that Hdj1 expressing cells have a distribution of larger prion aggregates as compared to the control strain. These size-shifts are consistent with a reduction in prion fragmentation in the Hdj1 samples, indicating that while Hdj1 is sufficient to replace Sis1, it may be less efficient than Sis1 in accomplishing this function. Notably, similar shifts are observable in some samples expressing Sis1-121 in **Figures 3B** **and S2**.

To assure that the maintenance of these strong [*PSI* ^+^] variants is due to Hdj1 expression only, we also examined these cells for Sis1 expression (**Figure S4**). A strong band for Sis1 is apparent in the control strain, but no comparable bands appear in any of the Hdj1-expressing strains maintaining strong [*PSI* ^+^] variants. To confirm that the loss of weak [*PSI* ^+^] variants in Hdj1-expressing cells is not due to differential protein expression in these strains, we next examined Hdj1 expression directly using a commercially available Hdj1 antibody. No band of the appropriate size was found in control cells lacking the Hdj1 expression plasmid, indicating that the antibody specifically recognizes Hdj1 and not Sis1 (**Figure 4B** **, lane 3**) Using this antibody, immunoblots of strains which lost the weak [*PSI* ^+^] variants and similar strains maintaining strong [*PSI* ^+^] variants indicated that Hdj1 expression levels are similar across all the strains examined (**Figure 4B**). Taken together, these results demonstrate that Hdj1 is capable of substituting for Sis1 in the maintenance of [*PSI* ^+^] in a prion-variant dependent manner that preferentially maintains strong, but not weak, [*PSI* ^+^] variants.

### Sis1 shuffling in a [*RNQ* ^+^]/[*PSI* ^+^]^Sc37^ strain allows for J-protein dependent prion selection and confirms mutually exclusive requirements for Sis1 among yeast prions

**Figure 5:**
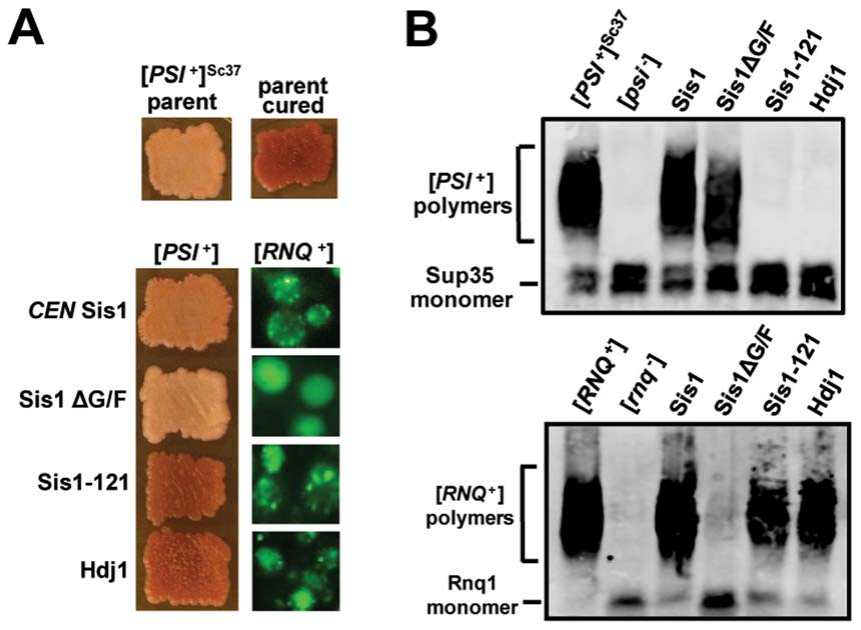
[*RNQ^+^*] and weak [*PSI^+^*]^Sc37^ are reciprocally maintained by Sis1ΔG/F and Sis1-121 when assayed simultaneously in the same yeast cells. (A and B) A *sis1*-Δ strain maintaining both [*RNQ* ^+^] and [*PSI* ^+^]^Sc37^ by expression of Sis1 from a *URA3*-marked plasmid ([*SIS1*-Sis1, *URA3*]) was transformed by plasmids expressing Sis1ΔG/F, Sis1-121 or Hdj1 and subjected to plasmid shuffling by growth on 5-FOA containing media. The maintenance of [*PSI* ^+^]^Sc37^ was assayed by colony color on rich media whereas the maintenance of [*RNQ* ^+^] was assayed by subsequent transformation of each shuffled strain by a Rnq1-GFP reporter plasmid ([*CUP1*-Rnq1-GFP]) followed by fluorescence microscopy analysis. (A) Color phenotype assays for [*PSI* ^+^]^Sc37^ or fluorescence patterns indicative of [*RNQ* ^+^] maintenance (punctate) or loss (diffuse) are shown for representative transformants (*n*≥6) for each construct following loss of the [*SIS1*-Sis1, *URA3*] plasmid. Cells transformed with full-length Sis1 (*CEN*-Sis1) are included as a positive control for prion maintenance throughout the shuffling and prion-detection procedures. Parental [*PSI* ^+^]^Sc37^ cells (parent) and cells cured by growth in the presence of GdnHCl (parent cured) are included as positive and negative controls for colony color. (B) Maintenance or loss of each prion in the cells from (A) was independently confirmed by semi-denaturing detergent agarose gel electrophoresis (SDDAGE). Detergent resistant Sup35 aggregates indicative of the presence of [*PSI* ^+^] (*upper image*) or detergent resistant Rnq1 aggregates indicative of the presence of [*RNQ* ^+^] (*lower image*) were resolved by SDDAGE and visualized by immunoblot analysis using antibodies specific for Sup35 or Rnq1, respectively. Control [*PRION* ^+^] and [*prion* ^−^] cells were included for comparison in each case.

The observation that weak [*PSI* ^+^] variants are maintained by Sis1-ΔG/F but not Sis1-121 or Hdj1 are of particular interest because they contradict the known Sis1 domain requirements of [*RNQ* ^+^], a prion which, until now, was considered to be more sensitive to Sis1 expression than either weak or strong [*PSI* ^+^] [24], [43]. An alternate explanation for the discrepancy between the Sis1 domain requirements between [*RNQ* ^+^] and weak [*PSI* ^+^] variants is that despite being examined in cells of the same genetic background, these two prions are not being examined within the same yeast cells, leaving open the possibility that an unanticipated polymorphism between our [*RNQ* ^+^] and [*PSI* ^+^]^Sc37^ tester strains, or an unrecognized difference in experimental conditions, is responsible for the disparate results. To address this issue, and to directly and unambiguously compare the requirements for these two prions, we mated our [*PSI^+^*]^Sc37^ strain to an otherwise isogenic [*RNQ^+^*] strain of opposite mating type in the W303 genetic background to create a new diploid strain that possessed both prions. Following sporulation and selection for the *sis1*::*LEU2* allele and the [*SIS1*-Sis1, *URA3*] plasmid, we isolated a [*RNQ^+^*]/[*PSI^+^*]^Sc37^ haploid strain again suitable for Sis1 plasmid shuffling. Following transformation of this new strain with our Sis1 and Hdj1 expression constructs and subsequent loss of the *URA3*-marked plasmid, confirmed again by both uracil auxotrophy and immunoblotting, the continued presence of [*PSI^+^*]^Sc37^ was again monitored by color assay while the presence of [*RNQ^+^*] was determined by fluorescence microscopy following a second transformation by a plasmid expressing an Rnq1-GFP chimera. [*RNQ^+^*] cells expressing Rnq1-GFP exhibit heterogeneous (punctate) fluorescence patterns as the fluorescent chimera is recruited into preexisting prion aggregates [23]. In a [*rnq* ^−^] cell the fluorescence is homogenously distributed about the cytoplasm (diffuse fluorescence) [23]. [*PSI* ^+^]^Sc37^ was again maintained by Sis1-ΔG/F but lost in the presence of either Sis1-121 or Hdj1 while results for [*RNQ* ^+^] matched those previously reported in the literature as expected: the prion was maintained by Sis1-121 and Hdj1 but lost in the presence of only Sis1-ΔG/F (**Figure 5A**) [41]. We again employed SDDAGE to confirm that both our color assay and GFP assay accurately report the aggregation states of the respective prion proteins and to examine any changes in aggregate size. Notably, SDDAGE analysis revealed that [*RNQ* ^+^] aggregates are larger in cells expressing only Sis1-121 or Hdj1, similar to the results observed for some strong [*PSI* ^+^] variants. More important however, for both prions, SDDAGE analyses unambiguously confirmed that the prions are lost or maintained in a reciprocal manner even when assayed in the same yeast cells (**Figure 5B**) demonstrating that these two prions have mutually exclusive requirements for Sis1 functions.

### Sis1's J-domain and G/M region are alone sufficient for cell viability and [*PSI* ^+^]^Sc37^ prion maintenance when expressed with a C-terminal tag

The results described above reaffirm that [*RNQ* ^+^] and strong [*PSI* ^+^] variants can be maintained minimally by co-expression (in *cis*) of only Sis1's J-domain and G/F regions (Sis1-121), yet this construct is insufficient for weak [*PSI* ^+^] propagation. Notably, this is the first time that a construct of Sis1 has been identified that separates the maintenance of cell viability from [*PSI* ^+^] prion propagation. The observation that Sis1-ΔG/F supports all variants of [*PSI* ^+^] examined herein raises the question: *What is the minimum Sis1 construct necessary for weak [PSI^+^] propagation*? Because of the apparent importance of Sis1's two glycine-rich regions and because Sis1-ΔG/F retains only G/M region, we speculated that this region may be of particular importance in the maintenance of weak [*PSI* ^+^] variants. To test this hypothesis, we first constructed a new expression construct for Sis1 which lacks the G/F region and ends with residue 171 (Sis1-171ΔG/F); this minimal construct consisted of only the J-domain of Sis1 and the G/M region (**Figure 1**). Although both Sis1-121 and Sis1-ΔG/F have been used as alternative minimal constructs for both prion maintenance and cell viability in the past, the minimal regions of Sis1 that are sufficient to support cell viability in the absence of the G/F region are also not known, so we first needed to confirm that this construct supports yeast cell viability independent of prion propagation. To avoid potential complications with prion-associated cytotoxicity, we first utilized cured ([*psi* ^−^]) versions of our plasmid shuffling strains. Despite successful isolation of transformants and repeated attempts at plasmid shuffling, no colonies formed on 5-FOA media indicating that this construct is unable to maintain cell viability as the sole form of Sis1. Because minimal J-domain expression constructs have been found to be active when expressed with a short, C-terminal trailer sequence like an HA-tag [56], we also investigated whether constructs expressing a few amino acids following the G/M region might be more active. We next constructed two new expression constructs for Sis1, again lacking the G/F region, but ending at either residue 171 or 206 and containing a random seven amino acid trailer sequence (Sis1-206ΔG/F* and Sis1-171ΔG/F*, **Figure 1**). Again, to avoid potential complications with prion-associated cytotoxicity, we first checked whether these constructs could support cell viability in cured versions of our plasmid shuffling strains. Following transformation, small numbers of slow-growing colonies formed on 5-FOA media indicating a successful replacement of the wild-type Sis1 plasmid and that both of the new Sis1 truncation constructs Sis1-206ΔG/F* and Sis1-171ΔG/F* maintain cell viability. These observations demonstrated that the critical functions of Sis1 may be accomplished by solely the expression of the J-domain in *cis* with the G/M region, as long as this region is not at the extreme C-terminus of the polypeptide. This result also confirms previous observations that the G/F and G/M domains have redundant functionality that is required for cell viability [40] [41], but now clarify that this function does not require co-expression of the C-terminal peptide binding or dimerization domains. Notably, we did not isolate many colonies on 5-FOA especially for Sis1-171ΔG/F*, indicating that, as one might expect, these minimal constructs are lacking when compared to full-length Sis1 and so it is difficult to isolate cells which preferentially lose the plasmid bearing the full-length construct.

**Figure 6:**
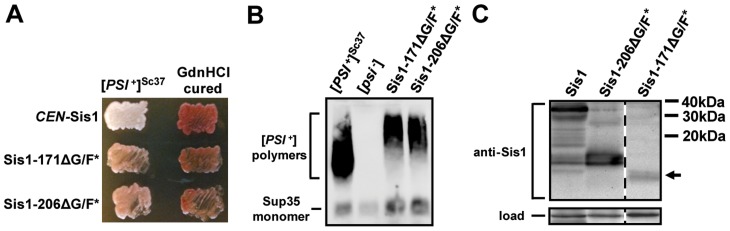
Sis1's J-domain and G/M region are sufficient to support cell viability and weak [*PSI* ^+^] propagation. (A) [*PSI* ^+^]^Sc37^ cells of the 74D-694 genetic background were transformed by plasmids expressing Sis1 truncations and subjected to plasmid shuffling. Color phenotype assays are shown for representative transformants following loss of the [*SIS1*-Sis1, *URA3*] plasmid. Cells expressing full-length Sis1 (*CEN*-Sis1) are used as a positive control for both colony color and the stability of the prion throughout the plasmid-shuffling procedure, whereas cells from each transformant were also cured by growth in the presence of GdnHCl and are included as negative controls for colony color. (B) Maintenance of [*PSI* ^+^]^Sc37^ in cells shown in (B) was also confirmed by semi-denaturing detergent agarose gel electrophoresis (SDDAGE). Detergent resistant Sup35 aggregates indicative of the presence of [*PSI* ^+^] were resolved by SDDAGE and visualized by immunoblot analysis using an antibody specific for Sup35. Control [*PSI* ^+^]^Sc37^ and [*psi* ^−^] cells were included for comparison. (C) The expression of Sis1 constructs, and the absence of wild-type Sis1 expression, in the cells from (A) was determined by subjecting cell lysates to SDS-PAGE followed by immunoblot analysis with Sis1-specific antibodies. Dotted lines separate lanes taken from different parts of the same gel. A band corresponding to Sis1-206ΔG/F* in lane two is clearly visible whereas a faint band corresponding to the approximate size of Sis1-171ΔG/F* is present in the right-most lane (*black arrow*). A band cross-reacting with the Sis1 antibody is shown as a loading control.

We next examined whether these same constructs could support [*PSI* ^+^] prion propagation by retransforming all of our prion tester strains. Interestingly, despite repeated attempts, we were able to isolate only a few viable colonies from 5-FOA media for any strains bearing strong [*PSI* ^+^] variants; however, SDSPAGE and immunoblotting confirmed that these colonies were still expressing full-length Sis1, despite the loss of the *URA3*-marked plasmid, indicating that a homologous recombination event had occurred during plasmid shuffling. In contrast, we were successful in isolating strains bearing the weak [*PSI* ^+^] variant [*PSI* ^+^]^Sc37^, though similar challenges with low viability and frequent cross-over events were also observed. **Figure 6** illustrates the results for these strains: both the Sis1-206ΔG/F* and Sis1-171ΔG/F* constructs maintained the pink colony color phenotype indicative of [*PSI* ^+^] as compared to parental-strain and cured-strain controls but with a notable change in color (**Figure 6A**). Likewise, SDDAGE analysis indicated prion maintenance, albeit with a severe shift in aggregate size toward higher molecular weights (**Figure 6B**). Immunoblotting with a Sis1 antibody confirmed that, unlike many other cells we isolated, these cells lacked full-length Sis1 expression (**Figure 6C**). Aggregate size and colony color can be causally related, that is, weaker [*PSI* ^+^] variants which are more difficult to fragment, tend to exhibit darker colony colors and larger prion aggregates than strong variants due to the decreased ability of large aggregates to sequester soluble Sup35. As such, both the dark color of the colonies and the high molecular weight bands observed here indicate that [*PSI* ^+^]^Sc37^ prion fragmentation is likely impaired in Sis1-206ΔG/F* or Sis1-171ΔG/F* expressing cells. Despite these apparent deficiencies in function, the observation that [*PSI* ^+^]^Sc37^ can be maintained at all by Sis1-171ΔG/F* in particular demonstrates that only the J-domain and G/M regions of Sis1 are fundamentally necessary for Sis1's function in weak [*PSI* ^+^] prion propagation and establish Sis1-171ΔG/F* as the new minimum construct for weak [*PSI* ^+^] maintenance for future experimentation.

#### Sis1's J-domain and G/F regions can maintain weak [*PSI* ^+^] variants only when expressed in *cis* with Sis1's C-terminal domains

**Figure 7:**
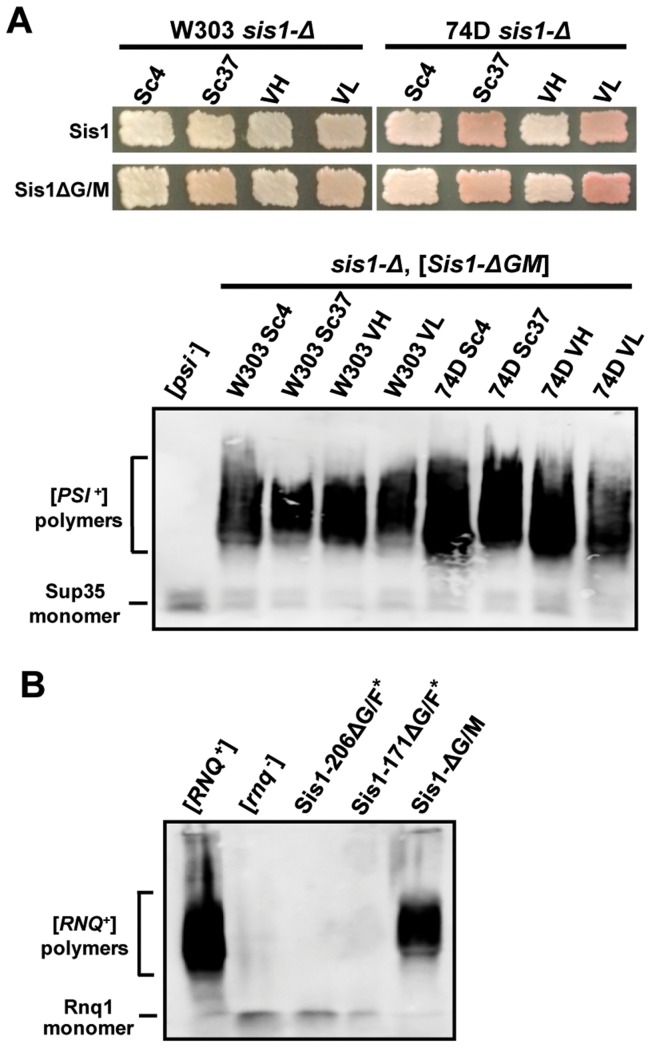
Sis1's J-domain and G/F region are sufficient to support the propagation of both strong and weak variants of [*PSI* ^+^] when additional C-terminal domains are present. (A) Yeast cells from two backgrounds (W303 and 74D-694) bearing strong or weak variants of [*PSI* ^+^] were transformed by a plasmid expressing Sis1Δ-G/M and subjected to plasmid shuffling. Color phenotype assays (*top*) are shown for representative transformants (*n*≥6 for each strain) following loss of the [*SIS1*-Sis1, *URA3*] plasmid. Cells expressing full-length Sis1 (Sis1) are used as a positive control for both colony color and the stability of the prion throughout the plasmid-shuffling procedure. Maintenance of each [*PSI* ^+^] variant in these cells was also confirmed by semi-denaturing detergent agarose gel electrophoresis (SDDAGE) (*bottom*). Detergent resistant Sup35 aggregates indicative of the presence of [*PSI* ^+^] were resolved by SDDAGE and visualized by immunoblot analysis using an antibody specific for Sup35. Control [*psi* ^−^] cells were included for comparison. (B) The maintenance or loss of [*RNQ* ^+^] in cells expressing only Sis1-171ΔG/F*, Sis1-206ΔG/F*, or Sis1-ΔG/M was also examined by transforming a W303 [*RNQ* ^+^] strain with plasmids bearing each Sis1 truncation and subjecting cells to plasmid shuffling. Cell lysates from resulting shuffled cells were the subjected to SDDAGE as described in (A) but blots visualized using antibody specific for Rnq1. Control [*RNQ* ^+^] and [*rnq* ^−^] cells were included for comparison.

The observations above demonstrate the J-domain and G/M regions are alone sufficient for weak [*PSI* ^+^] propagation. Since we have also found that all of our weak [*PSI* ^+^] variants were lost when only the J-domain and G/F regions are expressed (Sis1-121), we considered whether the G/M region might be essential for weak [*PSI* ^+^] propagation, or whether the G/F region could compensate when expressed in *cis* with the C-terminal domains. To test these alternate hypotheses, we next examined the ability of full-length Sis1 lacking only the G/M region (Sis1-ΔG/M, **Figure 1**) to maintain [*PSI* ^+^] variants. Because all strong variants were already shown to propagate in the presence of only Sis1-121, a shorter construct, these prions should be expected to also be supported by Sis1-ΔG/M. As expected, colony color and SDDAGE analyses verified that strong [*PSI* ^+^] variants persist in the presence of Sis1-ΔG/M, but also, surprisingly, Sis1-ΔG/M is also sufficient to maintain both of the weak variants we examined (**Figure 7A**). Once again, we replicated all results in both yeast genetic backgrounds (**Figure 7A**) and verified that all strains express only the Sis1-ΔG/M construct, rather than full-length Sis1, by immunoblotting (**Figure S5A**). Additionally, we investigated whether Sis1-ΔG/M exhibits a prion fragmentation defect by examining the prion aggregate size of each variant as compared to the same variant maintained by full-length Sis1 side-by-side (**Figure S5B**). No significant size changes were observed indicating that Sis1-ΔG/M is not detectably impaired in prion fragmentation.

As a final consideration to aid in drawing future comparisons, we verified whether each of the three new Sis1 constructs we analyzed herein (Sis1-206ΔG/F*, Sis1-171ΔG/F*, or Sis1-ΔG/M) are impaired in [*RNQ* ^+^] maintenance. As expected from prior work, because the G/F region is both necessary and sufficient for [*RNQ* ^+^] maintenance (in *cis* with the J-domain), Sis1-ΔG/M, but neither Sis1-206ΔG/F* nor Sis1-171ΔG/F*, maintained [*RNQ* ^+^] (**Figure 7B**) [41]. In total, these results demonstrate the G/F region is capable of substituting for the G/M region in weak [*PSI* ^+^] prion maintenance, but only when expressed together with the C-terminal domains of the protein. A summary of these results in total, along with all other known genetic interactions between the Sis1 constructs used in this study, and [*RNQ* ^+^] and [*PSI* ^+^] is included in Figure 1. Additional experimentation will be necessary to further clarify the role of these domains in prion biology.

## Discussion

The J-protein Sis1, an important component of the core prion-chaperone fragmentation machinery, possesses at least two distinct and prion-specific functionalities that arise from the combination of a functional J-domain and either of two glycine-rich domains, and only one of these functionalities has been evolutionarily maintained by the human homolog.

### Sis1 domain requirements are dependent upon the physical structure of the amyloid aggregate

In our analyses of Sis1 domain requirements by multiple [*PSI* ^+^] variants, we found a tremendous amount of consistency both between yeast genetic backgrounds and among prion variants of similar phenotypic strength. In contrast, Sis1 requirements differed greatly between so called ‘weak’ and ‘strong’ variants of [*PSI* ^+^], raising the question as to why these variants might exhibit such distinctions. Yeast prion variants are typically defined by phenotypic differences that arise from differences in the structure of the amyloid core [5] [45], [46], [57]. For two of the prions used here, [*PSI* ^+^]^Sc4^ and [*PSI* ^+^]^Sc37^, the relationship between phenotypic strength and amyloid structure is well-understood. [*PSI* ^+^]^Sc37^, the weaker variant, has a more extensive amyloid core with a greater number of residues involved and therefore a greater number of hydrogen bonds stabilizing the cross-beta structure [58]. As a result, [*PSI* ^+^]^Sc37^ aggregates are less easily fragmented *in vitro*, a characteristic that results in fewer heritable propagons per cell and a weaker prion phenotype *in vivo* [49] [46], [58]. It is reasonable to suggest, then, on the basis of our observations, that weak variants of [*PSI* ^+^] may require additional Sis1 intervention in order to allow prion fragmentation to keep up with cell division. Stein and True have recently shown that [*RNQ* ^+^] variants differ in Sis1 association suggesting that distinct prion variants may expose different regions of the protein to solvent for chaperone interactions [59]. Indeed, recent structural analyses by Frederick *et al.* have revealed that [*PSI* ^+^]^Sc4^ and [*PSI* ^+^]^Sc37^ fibers also differ in their interaction with Hsp104, possibly by virtue of a difference in the mobility of residues in the middle (M) domain of Sup35. That weak fibers bind better to Hsp104 is seemingly paradoxical, however as suggested in that investigation, this binding maybe non-functional and thereby obscure functional Hsp104 binding sites. It is reasonable to suggest, on the basis of our data, that these non-functional interactions may rather obscure Sis1 binding, which normally leads to the recruitment of Ssa and Hsp104 at partially unfolded sections of the protein. A combination of differences in structure of the prion aggregates, mobility of residues, and crowding by Hsp104 then would give rise to distinct binding interactions between Sis1 and weak [*PSI* ^+^] variants which might require specific portions of Sis1. Determining whether the removal of various domains impairs Sis1-binding *per se*, or the enabling of fragmentation after binding, will require additional co-aggregation experiments to distinguish.

Strong [*PSI* ^+^], on the other hand, appears to require very little from Sis1 by comparison. Despite now two direct investigations into the Sis1 domain requirements of strong [*PSI* ^+^], no single construct of Sis1 has been identified which can separate Sis1's role in maintaining cell viability from its role in strong [*PSI* ^+^] maintenance. These observations alone suggest that either Sis1 has more than one role in prion fragmentation, or that the distinctions between weak and strong variants are simply a matter of *stringency* in the requirement of a singular Sis1 function. Indeed, on the basis of prior data, an identical argument has been made to explain the discrepancies between the requirements for Sis1 between the prions [*PSI* ^+^] and [*RNQ* ^+^], however, as our data now suggest, the additional activities required by weak [*PSI* ^+^] variants and [*RNQ* ^+^] must be somehow distinct from one another.

### Minimal Sis1 constructs are prion-specific

As noted in the Results section, the patterns of prion loss that we observed are inconsistent with those that would be predicted if cytotoxicity was the driving factor in prion curing since curing occurred only in weak [*PSI* ^+^] variants with low propagon numbers. Likewise, the low propagon number of weak [*PSI* ^+^] variants is also insufficient alone to explain our results because another prion [*SWI* ^+^], which has even fewer propagons/cell than [*PSI* ^+^]^Sc37^, was found to be maintained by the same Sis1-121 expressing plasmid and in the same 74D-694 yeast strain during a previous investigation [25]. Additionally, the curing of [*PSI* ^+^] by Sis1 depletions was previously shown to be independent of Hsp104 overexpression [24]. Rather, the data presented here support the idea that Sis1's glycine-rich domains impart at least two distinct functionalities to the protein which prions require differentially. Specifically, the prions [*RNQ* ^+^] and [*PSI* ^+^]^Sc37^ can be selectively supported or lost in a reciprocal manner by replacement of wild-type Sis1 with a construct expressing only the J-domain and either the G/F region (Sis1-121) or the G/M region (Sis1-171ΔG/F*), respectively. *Do these regions impart prion-specific functions to Sis1?* In the case of [*RNQ* ^+^] and the G/F region, the answer appears to be ‘yes’, that is, to date no construct of Sis1 which lacks the G/F region has been found to support [*RNQ* ^+^] indicating that the G/F region is both necessary and sufficient in combination with the J-domain. However, with respect to weak [*PSI* ^+^] maintenance and the G/M region, the situation is complicated by functional overlap between the two regions that was first revealed in the context of cell viability; viability may be maintained by expression of only the J-domain and either glycine-rich region. While a construct consisting of only the J-domain and G/M region is sufficient to maintain weak [*PSI* ^+^], the construct Sis1-ΔG/M is also able to maintain all variants of [*PSI* ^+^] examined, indicating that there is also some functional overlap between the G/F and G/M domains in the context of prion maintenance but that the function(s) of these regions depends upon the context of the glycine-rich domain within the polypeptide that is expressed. It is interesting to note that this functional overlap appears to hierarchical, at least in this one instance; that is, the G/F region, in the context of the Sis1-ΔG/M construct, can substitute for the G/M region in weak [*PSI* ^+^] maintenance, but the reverse is not true for [*RNQ* ^+^], that is, G/M region is unable to substitute and support [*RNQ* ^+^] in the context of the Sis1-ΔG/F construct.

Notably, both minimal constructs produced noticeable increases in aggregate size in the respective prions they support, observations which would be consistent with either construct creating a small but noticeable defect in the efficiency of prion fragmentation. However, these size shifts were absent in cells bearing the longer constructs Sis1-ΔG/F and Sis1-ΔG/M which differ from the minimal constructs only by the addition of Sis1's C-terminal domains (*CTD1/2* and the dimerization domain (*DD*), **Figure 1**). This observation is interesting because, giving the ability of shorter constructs to maintain prions, the C-terminal domains of Sis1 are generally regarded as unimportant for prion maintenance. These observations indicate that the addition of the C-terminal domains to each respective minimal construct creates an observable change in aggregate size consistent with an increase the overall fragmentation of the prion aggregates which is similar to the full-length protein. This effect is not reproduced when only the first 35 residues of CTD1 are added back as in the Sis1-206ΔG/F* construct, demonstrating that is not simply a function of having additional amino acids at the C-terminal end of the glycine-rich domains. Nor does this effect appear to be due to expression issues, as at least for Sis1-121, protein levels are at least as great as the wild-type protein (**Figure 2C**). Sis1 is known to cycle in and out of the nucleus as part of spatial protein quality control and cytosolic misfolded protein-targeting for degradation by nuclear proteasomes [37] [36]. It is possible that our observations are largely affected by alterations in Sis1 localization then, particularly if Sis1 is sequestered to the nucleus when our minimal constructs are expressed. Intriguingly, one investigation found that movement of Sis1 into the nucleus was fully dependent upon its interaction with the sorting factors Btn2 and Cur1, which required the expression of Sis1 dimerization domain (*DD*, **Figure 1**), but not a functional J-domain or expression of CTD1/2 [37]. Considering that our minimal constructs (Sis1-121 and Sis1-171ΔG/F*) lack the dimerization domain while longer constructs (Sis1-ΔG/F and Sis1-G/M) maintain it, is highly unlikely that either the defects in fragmentation activity, or the distinctions between prions, revealed here are due to sequestration of Sis1 minimal constructs into the nucleus. Indeed, our experimental observations that single constructs have reciprocal effects on two prions expressed in the same cells further supports that assertion. Future Sis1 localization and co-aggregation experiments will help to further clarify not only this issue, but will also address the unanswered question of whether various mutant Sis1 constructs are deficient in prion-aggregate binding, or are competent for binding but fail to stimulate prion fragmentation. Additionally, sucrose gradient sedimentation may reveal unresolved changes in native aggregate size which may differ from changes in SDS-resistant aggregate size, and could lead to deficiencies in prion transmission to daughter cells. Regardless, these observations taken together indicate that while not essential, the C-terminal domains of Sis1 do contribute significantly to the ability of Sis1 to facilitate prion fragmentation, for both [*RNQ* ^+^] and [*PSI* ^+^].

### Prion-maintaining functions of Sis1 have been partially conserved in the human homolog

Our finding that the human homolog of Sis1, Hdj1, supports strong [*PSI* ^+^] strains conflicts with the observations of Kirkland *et al.* who found that a strong [*PSI* ^+^] variant was lost when Hdj1 was expressed in the absence of Sis1 [43]. These contradictory observations could be due to a difference in the yeast strain used, the specific prion variant examined, or due to a difference in amount of Hdj1 expression achieved in the experimental setup. The congruency of our other observations regarding strong [*PSI* ^+^] variants, both between variants in this study and with the observations made in that study make it unlikely that a difference in prion variant is to blame. The most likely reason for the discrepancy is that Hdj1 expression was driven by an exogenous promoter from a multicopy plasmid in our experiments, however we cannot, at present, rule out the possibility of an uncharacterized polymorphism between lab strains that may exist which effects prion-chaperone experimental results as we have observed similar phenomena in the past [42]. These conflicting observations underscore the benefit of confirming findings, when possible, in more than one genetic background and/or prion variant to control for strain- or variant-specific phenomena as well as protein expression levels.

One possible explanation for our observations regarding Hdj1's inability to support weak [*PSI* ^+^] is that perhaps Hdj1 is less active than Sis1 in prion fragmentation and the weak [*PSI* ^+^] variants examined here are simply more generally sensitive to reductions in Sis1 activity than [*RNQ* ^+^]. One means of testing this hypothesis would be examine the curing rates of these variants and [*RNQ* ^+^] upon Sis1 repression. Serendipitously, curing of both weak [*PSI* ^+^] variants examined here and [*RNQ* ^+^] have been examined within the same yeast genetic background (74D-694) under identical conditions during two previous investigations and exhibit typical sigmoidal curing curves upon Sis1 repression which may be compared by estimating the mid-point of curing, that is, the number of generations at which ∼50% of the cell population has been cured [42] [25]. In the case of the two weak [*PSI* ^+^] variants examined here, [*PSI* ^+^]^Sc37^ and [*PSI* ^+^]^VL^, ∼50% curing was attained at 17 and 22 generations, respectively [42]. Under identical conditions, the ∼50% curing mark for [*RNQ* ^+^] occurred at only 13 generations [25], a curing rate for [*RNQ* ^+^] which is consistent with previous estimations made in W303 background from GFP-counting data [24] [23]. Taken together, these observations indicate that [*RNQ* ^+^] is at least comparably sensitive to general reductions in Sis1 activity as either [*PSI* ^+^]^VL^ and [*PSI* ^+^]^Sc37^, if not more sensitive than both. Therefore, it is unlikely that Hdj1 maintains [*RNQ* ^+^] but not [*PSI* ^+^]^Sc37^ and [*PSI* ^+^]^VL^ simply due to differences in sensitivity to generic Sis1 activity, but rather suggest that Hdj1 lacks a distinct functionality of Sis1 that is specifically required by these weak variants.

Finally, we found that Hdj1 behaved similarly in our assays to the construct bearing only the J-domain and G/F region of Sis1 (Sis1-121), indicating that perhaps these regions are better conserved in the human protein. We have also observed that Hdj1 is incapable of substituting for Sis1 in the curing of [*PSI* ^+^] by Hsp104 overexpression, a phenomenon which also requires an unknown Sis1 function (*Hines J.K., unpublished observations*). It is plausible that the inability of Hdj1 to fully substitute for Sis1 in some biological functions stems from an inability to correctly partner with the yeast Hsp70s. Additional experiments co-expressing both Hdj1 and human Hsp70 will be necessary to further clarify the interpretation of these findings.

*Are Sis1 and Hdj1 amyloid recognition factors?* Several lines of evidence support this hypothesis. Sis1 is required for the propagation of all four yeast prions for which there is data ([*PSI* ^+^], [*RNQ* ^+^], [*URE3*], and [*SWI* ^+^]), and because it likely acts upstream of both Hsp70 and Hsp104, it is positioned to potentially be the first responding protein to direct chaperone activity toward amyloids [23] [21], [24], [25]. Sis1 is also found directly associated with other Q/N-rich proteins and polyglutamine aggregates in addition to yeast prions [60] [18], [36], [41], [61], and, perhaps most telling, a recent report revealed that Sis1 alone can ‘direct’ bacterial chaperones to maintain yeast prions [62]. If Sis1's role is indeed to ‘recognize’ amyloids *in vivo*, then understanding this functionality at the biochemical level would be of great interest. Perhaps even more intriguing would be to understand how one or the other of two short glycine-rich regions not only imparts Sis1's J-domain with the ability to maintain prions but imparts prion-*specific* maintenance. Additional work utilizing these new minimal constructs in combination with new insights about the human homolog will likely shed new light on this protein mystery in the near future.

## Methods

### Yeast strains and plasmids

Haploid *Saccharomyces cerevisiae* W303 and 74D-694 derived strains were used throughout. To create [*PSI* ^+^] strains competent for Sis1-plasmid shuffling, yeast strains bearing distinct [*PSI^+^*] variants from both backgrounds ([*PSI* ^+^] [*rnq* ^−^] [*p414-TETr-Sis1*] *sis1::LEU2 ade1-14 ura3-52 leu2-3*, *112 trp1-289 his3-200*) that were utilized in previous investigations for Sis1 repression experiments were transformed by a *URA3* marked plasmid expressing wild-type Sis1 (*p316-SIS1-Sis1*) [42]. Transformants were selected on synthetic media lacking uracil and then passaged on synthetic complete media containing 5-fluoroanthranilic acid (5-FAA) which counter-selects against the original *TRP1*-marked Sis1 expression plasmid (*p414-TETr-Sis1*). Strains were tested for [*PSI* ^+^] maintenance as well as uracil prototrophy and tryptophan auxotrophy prior to plasmid shuffling experiments. Additional W303 and 74D-694 strains bearing the strong variant [*PSI* ^+^]^93S^ were constructed by yeast lysate transformation in which recipient [*psi* ^−^] spheroplasts were co-transformed with cell extracts of the donor strain SL1293 (a gift from Susan Liebman) and the *URA3*-bearing Sis1 expression plasmid (*p316-SIS1-Sis1*). Transformants were then selected on media lacking uracil and candidate strains patched onto rich media to analyze prion status based on colony color. Prion status was verified by curability with GdnHCl and SDDAGE analysis as described in a subsequent section below.

To create a [*PSI* ^+^]/[*RNQ* ^+^] strain competent for Sis1-plasmid shuffling, the W303 [*PSI* ^+^]^Sc37^ plasmid shuffling strain described above ([*PSI* ^+^]^Sc37^ [*p316-SIS1-Sis1*] *sis1::LEU2 ade1-14 ADE2*) was mated to strain EAC Y639 ([*RNQ* ^+^] *ADE1 ade2-1*). Diploids were selected by adenine prototrophy and sporulated. Following tetrad dissection, pairs of haploids forming pink colonies from parental ditype tetrads were identified as *sis1::LEU2*, *ade1-14*, *ADE2*, [*PSI* ^+^] first by colony color and leucine prototrophy and then by the ability to convert to red-forming colonies upon GdnHCl treatment. Candidate haploids were then transformed by the plasmid *p413CUP1-RNQ1-GFP* and examined by fluorescence microscopy following selection on media lacking uracil. Strains exhibiting punctate fluorescence patterns, characteristic of [*RNQ* ^+^], which could be converted to a diffuse pattern upon GdnHCl treatment, were selected. Finally, the presence of both prions simultaneously was confirmed by semi-denaturing detergent agarose gel electrophoresis (SDDAGE) as described below in a later section.

Plasmids used in this study are based on the pRS series [63]. The gene fragments encoding Sis1-171ΔG/F (residues 1-70 and 122-171) or Sis1-206ΔG/F (residues 1-70 and 122-206) were amplified by polymerase chain reaction (PCR), introducing a 5′ *BamHI* site and 3′ *Sal1* site using plasmid *p313*-*SIS1*-Sis1-ΔG/F as the template. Linear insert was then digested with *BamHI* and *Sal1* and ligated (T4 DNA ligase) into pre-digested *p414-GPD*. Introduction of a random C-terminal seven amino acid tag (VDLESCN) was accomplished by site-directed mutagenesis PCR (Quikchange). Plasmid *p424-GPD*-*sis1-171ΔG/F* was likewise created by PCR amplification of *p313-SIS1*-*sis1-ΔG/F* to introduce sites for *EcoRI* and *SpeI*, upstream and downstream, respectively, followed by digestion with these enzymes and ligation into precut *p424-GPD*. All other plasmids, listed in **Table 1**, have been described elsewhere.

**Table 1:** Plasmids used in this study.

Plasmid	Promoter	Marker	Copy number	Source
*p413CUP1-RNQ1-GFP*	*CUP1*	*HIS3*	CEN, low	[23]
*p416CUP1-RNQ1-GFP*	*CUP1*	*URA3*	CEN, low	[40]
*p414TETr-SIS1*	*TETr*	*TRP1*	CEN, low	[23]
*p316SIS1-SIS1*	*SIS1*	*URA3*	CEN, low	[40]
*p313SIS1-SIS1*	*SIS1*	*HIS3*	CEN, low	[40]
*p313SIS1-sis1-ΔG/F*	*SIS1*	*HIS3*	CEN, low	[40]
*p324SIS1-sis1-ΔG/F*	*SIS1*	*TRP1*	2μ, high	[40]
*p314SIS1-sis1-121*	*SIS1*	*TRP1*	CEN, low	[40]
*p324SIS1-sis1-121*	*SIS1*	*TRP1*	2μ, high	[40]
*p314SIS1-sis1-ΔG/M*	*SIS1*	*TRP1*	CEN, low	[41]
*p424GPD-HDJ1*	*GPD*	*TRP1*	2μ, high	[41]
*p424GPD-sis1-171ΔG/F*	*GPD*	*TRP1*	2μ, high	This study
p414GPD-sis1-171ΔG/F*	*GPD*	*TRP1*	CEN, low	This study
p414GPD-sis1-206ΔG/F*	*GPD*	*TRP1*	CEN, low	This study

### SDS-PAGE and immunoblot analysis

Total protein extracts for SDS-PAGE were prepared by harvesting yeast cells in mid-log phase followed by vortexing in 1 M NaOH at 25°C. Cells were then spun at 13,500 rpm on a table-top centrifuge at 25°C and the supernatant was removed. Pellets were resuspended in sample buffer containing SDS and boiled for five minutes before resolving in a 12.5% polyacrylamide gel. The protein was transferred to nitrocellulose membrane at 1 A for 1 hour at 25°C in a tris-glycine/methanol buffer and probed with polyclonal antibodies specific to either Sis1 (a gift from the Craig lab) or Hdj1/DNAJB1 (Cayman Chemicals). *Western ladder* from New England Biolabs was used as a marker to detect relative protein sizes.

### Assays for cell growth and prion maintenance

To conduct plasmid shuffling experiments, *sis1*-Δ [*PRION* ^+^] cells expressing Sis1 from a *URA3*-marked plasmid were transformed by plasmids expressing either wild-type Sis1 or a Sis1-mutant protein and ∼10 transformants selected by growth on solid selective media. The action of the gene product of *URA3*, the enzyme orotidine-5′-phosphate decarboxylase, converts harmless 5-fluoro-orotic acid (5-FOA) into 5-flourouracil (5-FU), a chemotherapeutic agent which is toxic to dividing cells through its potent inhibition of thymidylate synthase. Subsequent growth on synthetic media containing 5-FOA counter-selects against the *URA3*-marked plasmid; only cells that stochastically lose the *URA3*-marked plasmid form colonies. Complete loss of the *URA3*-marked plasmid was then further confirmed by uracil auxotrophy. Following an additional passage on selective media to allow additional time for potential prion-loss, shuffled cells (6–10 transformants in each experiment) were examined for the maintenance of the prion by colony color on rich glucose media.

Propagon counting assays using GdnHCl and time course experiments utilizing *SIS1* under the control of the tetracycline-repressible promoter (*TETr-SIS1*) were conducted as previously described [24] [53], [64]. The presence or absence of [*PSI* ^+^] was confirmed by observation of colony color on glucose-based rich media YEPD (Teknova) where [*PSI* ^+^]-mediated aggregation of Sup35, a translation termination factor, causes read-through of the premature nonsense codon in the *ade1-14* mutant allele [65], [66]. Strains which are otherwise wild-type for adenine production appear pink or white in the presence of [*PSI* ^+^] or dark red in the absence of [*PSI* ^+^] due to the accumulation of a red intermediate when adenine production is blocked [67]. Cells were grown at 22°C for 3–6 days to allow color development prior to imaging. [*RNQ* ^+^] aggregates in cells were observed directly following transformation by a plasmid expressing Rnq1 fused to green-fluorescent protein (*p416CUP1-RNQ1-GFP*). [*RNQ* ^+^] cells can be easily distinguished from [*rnq^−^*] cells when examined under a microscope by characteristic punctuate or diffuse fluorescence patterns, respectively [23]. To create [*prion* ^−^] control strains, prion bearing cells were treated with the Hsp104 inhibitor GdnHCl (final concentration 4 mM) and grown in liquid culture with agitation for two days at 30°C to allow adequate cell divisions for prion curing.

Semi-denaturing detergent agarose gel electrophoresis (SDD-AGE), a method for resolving detergent resistant aggregates, was used to confirm the presence or absence of both [*RNQ* ^+^] and [*PSI* ^+^] and to determine relative aggregate size distributions [24], [68]. Briefly, cells were lysed using sterile glass beads by vortexing at 4°C. Following centrifugation at 4°C, cleared lysates were mixed with SDS loading buffer and incubated at 25°C for 7 minutes. Aggregates were resolved in a 1.5% (^w^/_v_) Tris-glycine (0.1% SDS) agarose gel (SeaKem Gold PFGE agarose) and protein was transferred to a nitrocellulose membrane at 1A for 1 hr at 22°C in a tris-glycine/methanol buffer. To visualize aggregates, membranes were blocked with 5% (^w^/_v_) milk and probed with antibodies specific for either Rnq1 or Sup35 (gifts from the Craig and Tuite labs, respectively).

## Supporting Information

Figure S1 Immunoblots of cells expressing Sis1ΔG/F. Sis1 protein expression levels in W303 strains bearing weak [*PSI* ^+^] variants ([*PSI* ^+^]^Sc37^, *left*, and [*PSI* ^+^]^VL^, *right*). Cell lysates were prepared from *sis1*-Δ cells expressing either wild-type Sis1 or Sis1ΔG/F from a plasmid and were subjected to SDS-PAGE followed by immunoblot analysis with anti-Sis1 specific antibodies. A band cross-reacting with the Sis1 antibody is shown as a loading control.
(TIF)

Figure S2 Sis1 domain requirements by [*PSI* ^+^] variants are indistinguishable between the W303 and 74D-694 yeast genetic backgrounds. (A) [*PSI* ^+^] cells of the 74D-694 genetic background bearing either (A) strong [*PSI* ^+^] variants ([*PSI* ^+^]^SC4^, [*PSI* ^+^]^STR^, [*PSI* ^+^]^VH^, and [*PSI* ^+^]^93S^) or (B) weak [*PSI* ^+^] variants (([*PSI* ^+^]^SC37^ and [*PSI* ^+^]^VL^) were transformed by plasmids expressing Sis1 truncations or deletions and subjected to plasmid shuffling. Color phenotype assays are shown for representative transformants (*n*≥6 for each plasmid) following loss of the [*SIS1*-Sis1, *URA3*] plasmid. Parental [*PSI* ^+^] cells for each variant (parent) and cells cured by growth in the presence of GdnHCl (cured) are included as positive and negative controls for colony color. Cells expressing full-length Sis1 (Sis1) from a plasmid were used as a positive control for the stability of the prion throughout the plasmid-shuffling procedure. For clarity, images taken from different parts of the same plate have been arranged in columns. (C) Maintenance or loss of weak [*PSI* ^+^] variants in cells shown in (B) was also confirmed by semi-denaturing detergent agarose gel electrophoresis (SDDAGE). Detergent resistant Sup35 aggregates indicative of the presence of [*PSI* ^+^] were resolved by SDDAGE and visualized by immunoblot analysis using an antibody specific for Sup35. Control [*PSI* ^+^] and [*psi* ^−^] cells for each variant were included for comparison. (D) Maintenance of strong [*PSI* ^+^] variants ([*PSI* ^+^]^SC4^, [*PSI* ^+^]^STR^, [*PSI* ^+^]^VH^, and [*PSI* ^+^]^93S^) in 74D-694 cells shown in (A) was also confirmed by semi-denaturing detergent agarose gel electrophoresis (SDDAGE). Detergent resistant Sup35 aggregates indicative of the presence of [*PSI* ^+^] were resolved by SDDAGE and visualized by immunoblot analysis using an antibody specific for Sup35. Control [*PSI* ^+^] and [*psi* ^−^] cells for each variant were included for comparison.
(TIF)

Figure S3 Propagon counting assays for the six [*PSI* ^+^] variants used in this study. Cells were cultured in rich liquid media with aeration for at least four generations in log growth before the addition of 4 mM GdnHCl (generation = 0). Cells were maintained in log growth at 30°C for 14–16 generations and plated to YPD at a density of 200–300 cfus per plate approximately once per generation. Colonies were allowed to form at 22°C for 4–5 days for color development and were counted and data modeled as previously described [24] [53], [64]. Strong [*PSI* ^+^] variants (STR, Sc4, VH, 93S) were indistinguishable on the basis of these data and are shown fit to a model positing 300 propagons/cell. The weak [*PSI* ^+^] variants Sc37 and VL are shown with a fit-lines positing 90 and 75 propagons/cell, respectively.
(TIF)

Figure S4 Immunoblot of cells expressing Hdj1 visualized with antibody specific for Sis1. Cell lysates of *sis1*-Δ cells expressing Hdj1 from a plasmid and maintaining strong [*PSI* ^+^] variants were resolved by SDS-PAGE and visualized by immunoblotting with a Sis1-specific antibody that does not cross-react with Hdj1. Molecular weight markers and a lysate of a control strain expressing only full-length Sis1 were loaded into lanes one and two, respectively. A band cross-reacting with the Sis1 antibody in all lanes is shown as a loading control (*black arrow*).
(TIF)

Figure S5 Biochemical assays confirm Sis1-ΔG/M expression and prion aggregate size. (A) Cells shown in **Figure 7A** were lysed and subjected to SDS-PAGE followed by immunoblot analysis using Sis1 specific antibodies. (B) Plasmid shuffled strains containing Sis1-ΔG/M exhibit no clear size differences in Sup35 aggregates than those expressing wild-type Sis1. Cells from **Figure 7A** were lysed and subjected to SDDAGE followed by immunoblot analysis using anti-Sup35 specific antibodies. In this case, adjacent lanes were loaded with lysates from cells of the same background, and bearing the same prion variant, and expressing either full-length Sis1 or Sis1-ΔG/M side-by-side to enable direct size comparisons. Control [*psi* ^−^] cells were included for comparison.
(TIF)
