# Supplementary figures and images for: Functional Diversification of Hsp40: Distinct J-Protein Functional Requirements for Two Prions Allow for Chaperone-Dependent Prion Selection
Source: PLoS Genet. 2014 Jul 24;10(7):e1004510. doi: 10.1371/journal.pgen.1004510 (PMC4109904; doi:10.1371/journal.pgen.1004510)

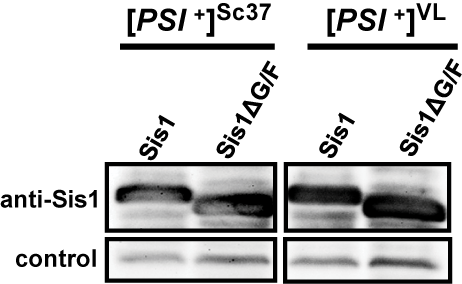

Supplement: Figure S1 — Immunoblots of cells expressing Sis1ΔG/F. Sis1 protein expression levels in W303 strains bearing weak [PSI +] variants ([PSI +]Sc37, left, and [PSI +]VL, right). Cell lysates were prepared from sis1-Δ cells expressing either wild-type Sis1 or Sis1ΔG/F from a plasmid and were subjected to SDS-PAGE followed by immunoblot analysis with anti-Sis1 specific antibodies. A band cross-reacting with the Sis1 antibody is shown as a loading control. (TIF) [file pgen.1004510.s001.tif]

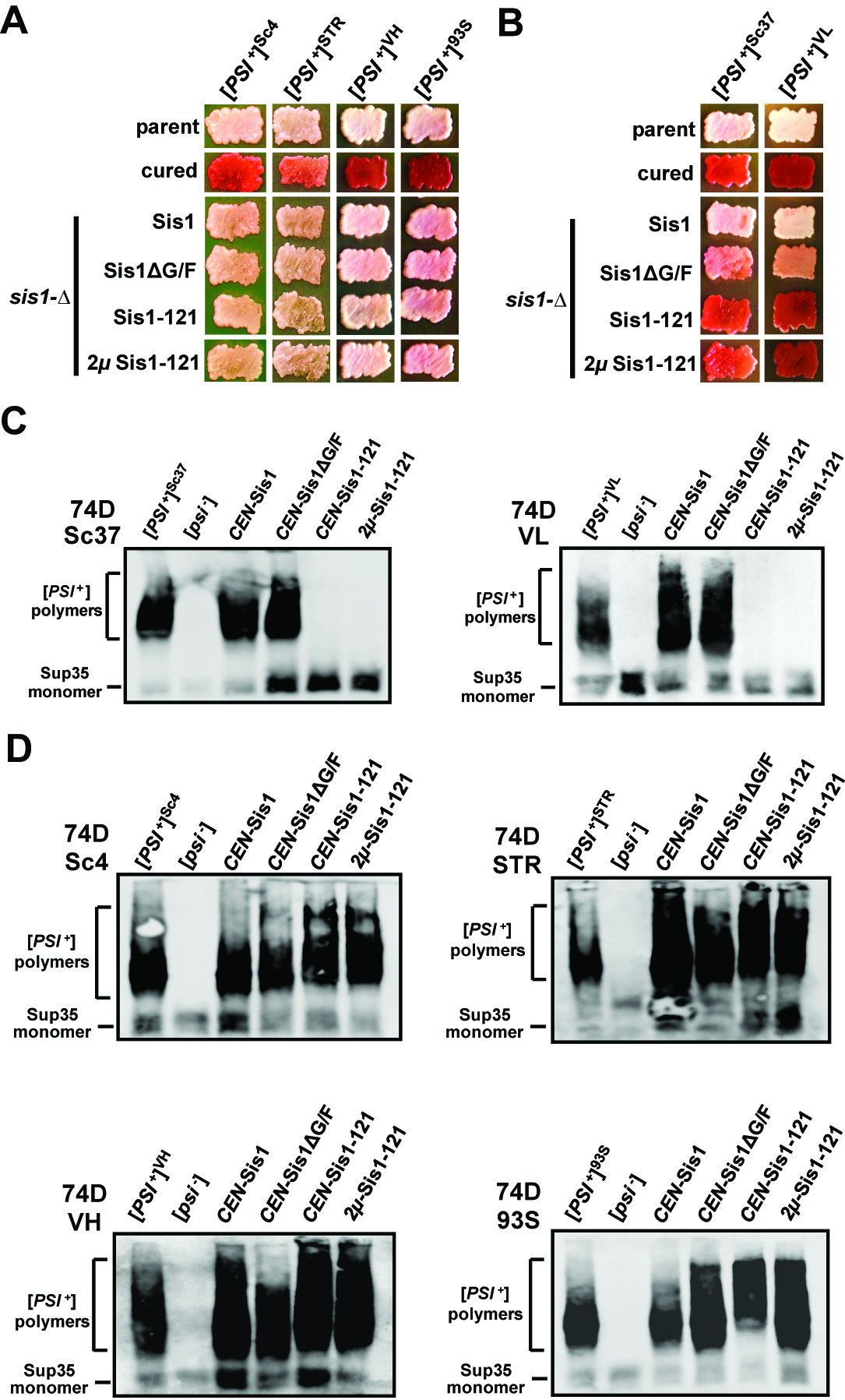

Supplement: Figure S2 — Sis1 domain requirements by [PSI +] variants are indistinguishable between the W303 and 74D-694 yeast genetic backgrounds. (A) [PSI +] cells of the 74D-694 genetic background bearing either (A) strong [PSI +] variants ([PSI +]SC4, [PSI +]STR, [PSI +]VH, and [PSI +]93S) or (B) weak [PSI +] variants (([PSI +]SC37 and [PSI +]VL) were transformed by plasmids expressing Sis1 truncations or deletions and subjected to plasmid shuffling. Color phenotype assays are shown for representative transformants (n≥6 for each plasmid) following loss of the [SIS1-Sis1, URA3] plasmid. Parental [PSI +] cells for each variant (parent) and cells cured by growth in the presence of GdnHCl (cured) are included as positive and negative controls for colony color. Cells expressing full-length Sis1 (Sis1) from a plasmid were used as a positive control for the stability of the prion throughout the plasmid-shuffling procedure. For clarity, images taken from different parts of the same plate have been arranged in columns. (C) Maintenance or loss of weak [PSI +] variants in cells shown in (B) was also confirmed by semi-denaturing detergent agarose gel electrophoresis (SDDAGE). Detergent resistant Sup35 aggregates indicative of the presence of [PSI +] were resolved by SDDAGE and visualized by immunoblot analysis using an antibody specific for Sup35. Control [PSI +] and [psi −] cells for each variant were included for comparison. (D) Maintenance of strong [PSI +] variants ([PSI +]SC4, [PSI +]STR, [PSI +]VH, and [PSI +]93S) in 74D-694 cells shown in (A) was also confirmed by semi-denaturing detergent agarose gel electrophoresis (SDDAGE). Detergent resistant Sup35 aggregates indicative of the presence of [PSI +] were resolved by SDDAGE and visualized by immunoblot analysis using an antibody specific for Sup35. Control [PSI +] and [psi −] cells for each variant were included for comparison. (TIF) [file pgen.1004510.s002.tif]

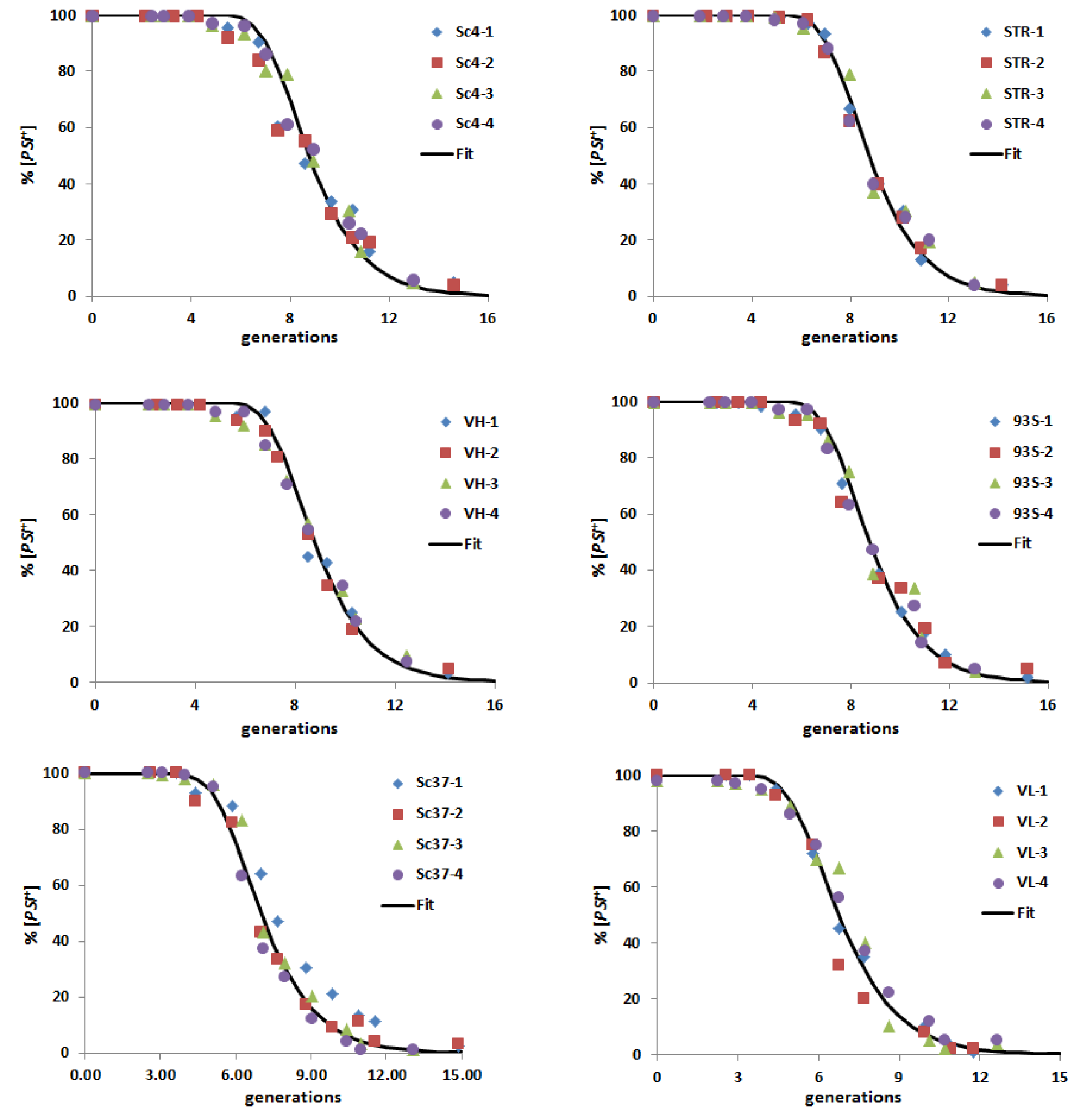

Supplement: Figure S3 — Propagon counting assays for the six [PSI +] variants used in this study. Cells were cultured in rich liquid media with aeration for at least four generations in log growth before the addition of 4 mM GdnHCl (generation = 0). Cells were maintained in log growth at 30°C for 14–16 generations and plated to YPD at a density of 200–300 cfus per plate approximately once per generation. Colonies were allowed to form at 22°C for 4–5 days for color development and were counted and data modeled as previously described [24] [53], [64]. Strong [PSI +] variants (STR, Sc4, VH, 93S) were indistinguishable on the basis of these data and are shown fit to a model positing 300 propagons/cell. The weak [PSI +] variants Sc37 and VL are shown with a fit-lines positing 90 and 75 propagons/cell, respectively. (TIF) [file pgen.1004510.s003.tif]

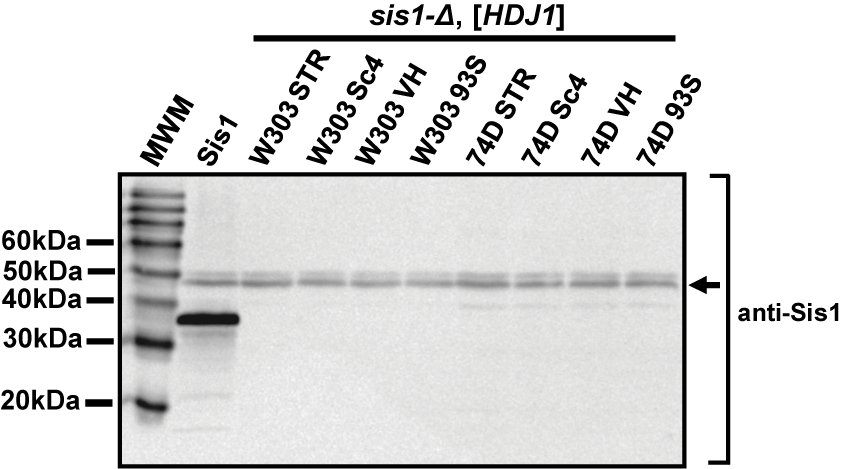

Supplement: Figure S4 — Immunoblot of cells expressing Hdj1 visualized with antibody specific for Sis1. Cell lysates of sis1-Δ cells expressing Hdj1 from a plasmid and maintaining strong [PSI +] variants were resolved by SDS-PAGE and visualized by immunoblotting with a Sis1-specific antibody that does not cross-react with Hdj1. Molecular weight markers and a lysate of a control strain expressing only full-length Sis1 were loaded into lanes one and two, respectively. A band cross-reacting with the Sis1 antibody in all lanes is shown as a loading control (black arrow). (TIF) [file pgen.1004510.s004.tif]

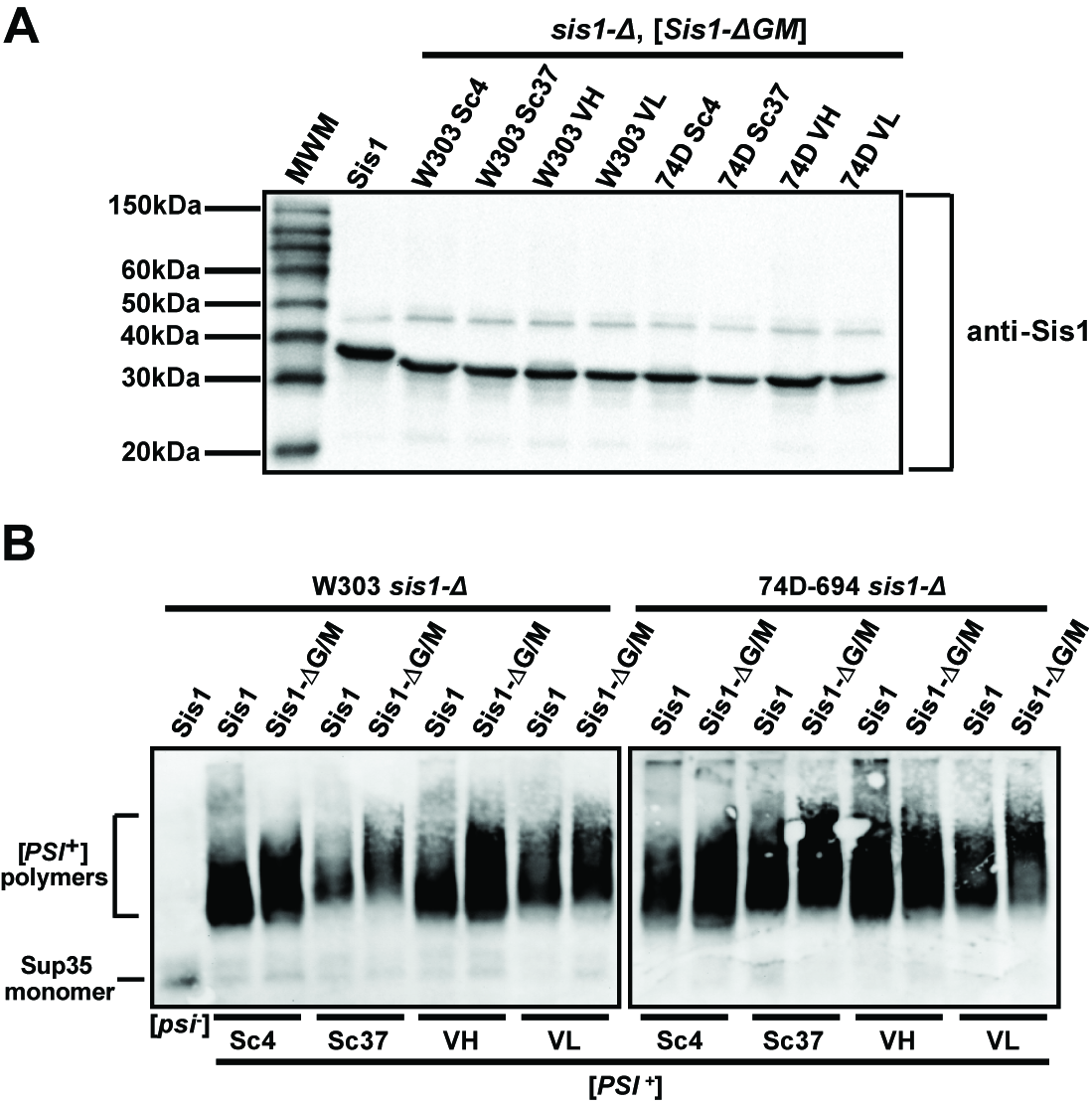

Supplement: Figure S5 — Biochemical assays confirm Sis1-ΔG/M expression and prion aggregate size. (A) Cells shown in Figure 7A were lysed and subjected to SDS-PAGE followed by immunoblot analysis using Sis1 specific antibodies. (B) Plasmid shuffled strains containing Sis1-ΔG/M exhibit no clear size differences in Sup35 aggregates than those expressing wild-type Sis1. Cells from Figure 7A were lysed and subjected to SDDAGE followed by immunoblot analysis using anti-Sup35 specific antibodies. In this case, adjacent lanes were loaded with lysates from cells of the same background, and bearing the same prion variant, and expressing either full-length Sis1 or Sis1-ΔG/M side-by-side to enable direct size comparisons. Control [psi −] cells were included for comparison. (TIF) [file pgen.1004510.s005.tif]
